# Characterization and validation of a bone metastatic castration-resistant prostate cancer model as a nanomedicine evaluation platform

**DOI:** 10.7150/thno.123005

**Published:** 2026-01-01

**Authors:** Antoni Serrano-Martí, Ana Armiñán, Inmaculada Conejos-Sánchez, Daniela Mittermüller, Shang-Wei Li, Paula Tenhaeff Lackschewitz, Esther Roselló-Sastre, Matthias Gunzer, Horacio Cabral, María J. Vicent

**Affiliations:** 1 Polymer Therapeutics Laboratory, Príncipe Felipe Research Center (CIPF), Valencia, Spain.; 2 Centro de Investigación médica en Red Cáncer (CIBERONC), Madrid, Spain.; 3 Institute for Experimental Immunology and Imaging, University Hospital, University of Duisburg-Essen, Essen, Germany.; 4 Institute for Virology, University Hospital, University of Duisburg-Essen, Essen, Germany.; 5 Leibniz-Institut für Analytische Wissenschaften - ISAS - e.V., Dortmund, Germany.; 6 Department of Bioengineering, Graduate School of Engineering, The University of Tokyo, Tokyo, Japan.; 7 Department of Pathology, Consorcio Hospital General Universitario de Valencia, Valencia, Spain.

**Keywords:** prostate cancer, bone metastasis, nanomedicine, polymer-drug conjugates, docetaxel

## Abstract

**Rationale:** Bone metastases - common in metastatic castration-resistant prostate cancer (mCRPC) - lead to severe complications and currently suffer from limited therapeutic options. Poor solubility, systemic toxicity, and therapeutic resistance hamper conventional approaches, such as docetaxel (Dtx) treatment. Nanomedicine-based strategies - including polymer-drug conjugates - can help overcome said limitations through enhanced tumor targeting and reduced unwanted side effects in healthy tissues.

**Methods:** An intratibial bone mCRPC mouse model - used to recapitulate tumor growth and microenvironmental dynamics - was developed and characterized. A poly-L-glutamic acid (PGA)-Dtx) conjugate synthesized to enhance Dtx delivery and efficacy was also characterized in terms of size, zeta potential, drug loading, and pH-dependent release. *In vivo* evaluations included tumor growth monitoring by bioluminescence imaging, cathepsin K activity from tumor by fluorescence imaging, bone damage evaluation by micro-computed tomography, tumor vasculature by light-sheet fluorescent microscopy, cell population at tumor site by histology, modulation of blood cell populations by tumor and treatment by hematology, and biodistribution of PGA-Dtx using fluorescent imaging and intravital microscopy.

**Results:** Our intratibial bone mCRPC model supported reliable tumor establishment, progressive osteolytic damage and vascularization, and systemic inflammation. PGA-Dtx displayed optimal properties (6.6 nm size, -24.1 mV zeta potential, 3.3 mol % drug loading) and supported lower but sustained Dtx release at acidic pH. The enhanced tumor accumulation following PGA-Dtx administration significantly suppressed tumor growth *in vivo*, normalized cathepsin K activity levels, and reduced bone damage while avoiding the systemic toxicity associated with free Dtx.

**Conclusions:** Our intratibial bone mCRPC mouse model provides a robust platform for studying PCa bone metastases and evaluating nanomedicine efficacy. PGA-Dtx displays promise as a safe and effective therapy for mCRPC, offering improved drug delivery and reduced systemic side effects, which supports the translational potential of polymer-drug conjugates in mCRPC management.

## Introduction

Prostate cancer (PCa) typically begins as a localized disease but progresses to castration-resistant prostate cancer (CRPC) within five years in ∼20% of cases, with affected patients suffering an average survival rate of 2-3 years 1. CRPC progression typically leads to metastatic (m)CRPC within a few years; this disease stage has limited treatment options and poor prognosis, with 65-75% of patients developing bone metastases, which worsen outcomes through complications like bone pain, fractures, hypercalcemia, and nerve compression 2.

Various *in vitro* preclinical models have been developed to study bone mCRPC (including three-dimensional (3D) models); however, they do not fully recapitulate the characteristics of the *in vivo* scenario. Bone implant models, transgenic mouse models, and patient-derived xenografts have been developed to study PCa bone metastasis *in vivo*; however, low levels of bone metastasis, biomechanical mismatch, loss of tumor heterogeneity, and the poor statistical power associated with small sample sizes represent challenges 3. Direct injection models vary in utility but face limitations regarding replicating bone metastasis. Subcutaneous models do not recapitulate PCa progression well and rarely metastasize to the bone; bone metastasis rarely arises in orthotopic models. Tail vein injection models represent an excellent means of studying metastatic processes from circulation to a metastatic niche, but they primarily seed lung metastasis and rarely seed bone metastasis. Finally, intracardiac models can induce bone metastasis but present limitations regarding the number of cells injected and often induce lethal cancers 3. The intraosseous model, widely used to study the effects of PCa on bone and important to clinical advances in this area, supports the study of tumor microenvironment (TME) interactions and therapeutic responses but lacks representation of early metastatic stages. Notably, ∼10% of newly diagnosed PCa patients present with bone metastasis when the early stages of the metastatic process have already occurred 4. Despite this limitation, the intraosseous model provides high tumor initiation rates and controlled tumor placement, making it ideal for studying treatments for established bone metastases.

A deeper understanding of PCa has driven the development of nanomedicine-based treatment strategies 5. Although we still lack FDA-approved PCa nanomedicines, the evaluation of polymeric nanomedicines, including Opaxio 6 and Osteodex 7, and liposomal-based strategies (e.g., Doxil, Leuvectin, and MM-310), have provided proof that this approach may address the challenges associated with treating PCa bone metastasis 8. Polymer therapeutics 9, and in particular polypeptide-based nanoconjugates (PDCs) - distinguished by a covalent linking moiety that connects the water-soluble multivalent polymeric carrier and the bioactive cargo - provide clinical advantages 10. Adjusting linker properties can optimize drug delivery and therapeutic outcomes, while inherent linker responsivity to tumor-specific signals can improve treatment localization and effectiveness and reduce systemic toxicity. Linkers also support the integration of multiple functionalities into a single platform, enabling multifunctional nanoconjugates that boost therapeutic efficacy through synergistic effects and overcome multidrug resistance 9, 10. Furthermore, this advantage supports the integration of imaging, diagnostics, and drug delivery into a single platform. Developing "theranostic" approaches that combine therapeutic and diagnostic capabilities have enabled real-time tumor progression and treatment response monitoring 11.

Of note, within PDCs, Opaxio (PGA-Paclitaxel) enhanced water solubility, antitumor efficacy, and reduced toxicity 12. In preclinical models, a single intravenous dose of PGA-paclitaxel led to complete tumor regression and showed prolonged plasma half-life, increased tumor uptake, and limited conversion to free paclitaxel in the bloodstream or tumor tissue within 144h after intravenous injection 13. It caused more extensive tumor necrosis than paclitaxel, suggesting additional pharmacological effects. Reaching phase IV trials, PGA-paclitaxel showed improved solubility, stability, and potential to overcome resistance 14. However, it was not FDA-approved due to factors including economic and patent-related issues 15.

A thorough understanding of the specific characteristics of metastatic PCa remains essential to developing effective nanomedicine strategies. Mouse models have been instrumental in elucidating the biological processes involved in PCa bone metastasis, allowing the investigation of the TME and the effects of treatments in a controlled setting 16. However, the translation of findings from models to clinical settings remains a hurdle, as many nanomedicines have not demonstrated efficacy in human trials despite promising results in preclinical studies 17.

Here, we optimized and exhaustively characterized a mouse model of bone mCRPC based on intratibial injection and evaluated the model's utility regarding the evaluation of nanomedicines to i) better understand the bone metastasis microenvironment and associated processes in PCa, ii) improve the rational development of nanomedicine-based therapies, and iii) enhance clinical translation.

## Materials and Methods

The Supplementary Information contains all materials and a detailed description of all methods employed; a summary of the key techniques used is provided below.

### Development of a bone mCRPC model

PC3-Luc cells were harvested at 70-80% confluency, mixed with Matrigel and RPMI-1640 media, and injected into anesthetized mice tibias within 1 h to maintain viability. Tumor growth was monitored biweekly for six weeks via *in vivo* bioluminescence imaging using the IVIS® Spectrum. Luciferin was administered for visualization, and tumor luminescence was analyzed using Living Image® software.

### *In vivo* analysis of cathepsin K release/activity in mouse tibias

Cathepsin K activity was monitored *in vivo* using the IVISense™ Cat K 680 FAST Fluorescent Probe in healthy and tumor-injected tibias for six weeks, which remains inactive until cleaved by Cathepsin K to produce fluorescence (λex/λem = 675/693 nm). Baseline fluorescence images were captured before injection, with 1.6 nmol of the probe administered intravenously each week. Fluorescent images were acquired 6 h post-injection following the manufacturer's guidelines. Non-tumor-inoculated right tibias served as healthy controls.

### *Ex vivo* microtomography of mouse tibias

*Ex vivo* micro-computed tomography (µCT) imaging of healthy and tumor-affected tibias was performed weekly for six weeks using an MRS*CT Benchtop system (MR-Solutions) operating at 46-60 kVp and 1 mA, with a spatial resolution of 50 µm, and a voxel size of 25 µm. Images were acquired at 40 kVp, 0.5 mA, and a 75 ms exposure time, with a 64.46 mm field of view, taking 7.5 min per scan. Images were reconstructed using the Iterative Least Squares algorithm with beam-hardening correction for soft tissue, and Hounsfield Unit values were calculated.

### Light-sheet fluorescence microscopy studies

After tumor inoculation, mice were divided into four groups based on similar luminescence signals to assess vascular development over four weeks. Each week, one group received an intravenous injection of 10 µg anti-CD31 fluorescent antibody (CD31-AF647), which is highly expressed on endothelial cells, lining in the interior of blood vessels, before euthanasia. Mice were perfused with PBS/EDTA and fixed with 4% PFA. Tibias were post-fixed, dehydrated, and cleared using ethyl cinnamate for deep tissue imaging. Whole tibias were imaged with a LaVisionBioTec (Miltenyi) UltraMicroscope Blaze, a 200 mW white light laser (480 - 2.400 nm) and excitation-emission bandpass filter combination of 630/30:680/30 for detection of AF647 and 488/20:525/30 for detection of tibia tissue by green signal autofluorescence. Images were taken with the 4x magnification dipping objective and analyzed with IMARIS software.

To evaluate the effect of PGA-Dtx treatment, tumor-inoculated mice were divided into a vehicle-treated group (PBS) and a treatment group receiving 7.5 mg/kg PGA-Dtx twice weekly for four weeks. The same CD31-AF647 staining, imaging, and analysis protocol was followed at the study endpoint.

### Analysis of the enhanced permeability and retention effect during tumor development

The enhanced permeability and retention (EPR) effect was assessed weekly until week 4 by intravenously injecting mice with 50 mg/kg Dextran, Texas Red™ (40 kDa). Mice were euthanized 1 h post-injection, the tibias were extracted, and fluorescence intensity (λex/λem = 595/615 nm) was measured using the IVIS® Spectrum and then quantified.

### Study of PGA-Dtx-Cy5.5 stability in circulation by intra-vital microscopy

PGA-Dtx-Cy5.5 stability in circulation was evaluated using intravital microscopy (IVM) in healthy NOD.CB17-*Prkdc^scid^*/NCrHd mice after the intravenous injection (7.5 mg/kg). Imaging was performed with a Nikon A1R confocal laser scanning microscope using a 20x objective, a 640 nm diode laser, and a 700/75 nm emission filter. Mice were anesthetized with isoflurane and catheterized via the lateral tail vein for injection. The ear-lobe dermis was imaged non-invasively under a coverslip with immersion oil. Data were recorded in video mode for 6 h post-injection and analyzed using NIS-Elements software (Nikon, Tokyo, Japan).

### Analysis of PGA-Dtx-Cy5.5 tumor accumulation using the Xenogen IVIS® Spectrum

PGA-Dtx-Cy5.5 accumulation in tumors was monitored weekly using the IVIS® Spectrum. Mice received an intravenous injection of 7.5 mg/kg PGA-Dtx-Cy5.5, and fluorescence images were captured at λex/λem = 683/703 before injection (baseline) and at 1, 4-, 24-, 48-, and 96-h post-injection. The fluorescence signal was analyzed using the Living Image® (64-bit) software.

### Analysis of PGA-Dtx-Cy5.5 tumor accumulation using intra-vital microscopy

PC3-Luc-GFP tumor cells were injected into the left tibia of NOD.CB17-*Prkdc^scid^*/NCrHd mice (6-8 weeks old), and tumor engraftment was monitored using the IVIS® Spectrum. Mice were divided into four groups based on luminescence signals to assess PGA-Dtx-Cy5.5 accumulation weekly. One group per week underwent IVM to analyze the tumor microenvironment (TME; vascularity and permeability).

To evaluate PGA-Dtx-Cy5.5 effects on the TME in real time, tumor-bearing mice were split into two groups: one vehicle-treated group (PBS) and another receiving 7.5 mg/kg PGA-Dtx twice weekly for four weeks. At the endpoint, both groups underwent IVM after PGA-Dtx-Cy5.5 injection.

For IVM, mice were anesthetized, their tibias surgically exposed while keeping vessels intact, and stabilized under the microscope. The tumor area was identified using GFP fluorescence (λex/λem = 395/509), and PGA-Dtx-Cy5.5 (λex/λem = 683/703) was intravenously injected to observe vascular passage and tumor extravasation over one h. Data were recorded in video mode and analyzed with NIS-Elements software (Nikon, Tokyo, Japan).

### PGA-Dtx conjugate synthesis

Linear PGA (0.75mmol, 1 equivalent) was dissolved in anhydrous DMF in a two-neck round bottom flask fitted with a stir bar under an inert atmosphere. Once completely dissolved, a catalytic amount of DMAP and EDC (0.23 mmol, 0.30 equivalents) was added to the reaction mixture and left stirring for 20 min. Then, Dtx (0.23 mmol, 0.30 equivalents) was added to the reaction, and the pH was adjusted to 8.0 with N,N-diisopropylethylamine (DIEA). The reaction was left to proceed with stirring at room temperature for 24 h. The product was purified by precipitation in cold diethyl ether, and the white powder obtained was dried under a vacuum. After drying, PGA-Dtx was dissolved in Milli-Q water, converted to its salt form by adjusting the pH to 8.0 with 0.5 M NaHCO₃, purified using a Vivaspin™ 3000 MWCO filter to remove excess salts and free drug, washed with ~100 mL Milli-Q water, and then freeze-dried.

### Total drug loading and free drug content

*Total Drug loading* was determined using ultraviolet-visible (UV-VIS) spectroscopy. A Dtx calibration curve was prepared in a methanol:water mixture, with absorbance measured at 227 nm. Conjugates (sodium salt form) were dissolved in double-distilled water, and absorbance spectra were recorded under the same conditions. PGA-Dtx drug loading (% wt) was calculated by interpolating absorbance values in the calibration curve. Measurements were performed using a JASCO V-630 spectrophotometer at 25°C with 1.0 cm quartz cells, a 1 nm spectral bandwidth, and three accumulations per sample. *Free Drug content:* To quantify the free drug (Dtx) in the PGA-Dtx conjugate, 3 mg of the sample was dissolved in 500 µL of LC-MS-grade methanol, vortexed, and centrifuged. The supernatant was filtered and analyzed using LC-MS/MS with an ExionLC and AB Sciex QTRAP 4500 system. Detection employed paclitaxel (1 µg/mL) as an internal standard, with specific ion transitions for Dtx and Ptx under positive electrospray ionization. LC-MS conditions included a 70% acetonitrile mobile phase with 0.05% TFA, a C18 column at 40 °C, and a 10 µL injection volume. The resulting free drug content was found to be 0.23% w/w relative to the total drug.

### Nuclear magnetic resonance

Proton nuclear magnetic resonance (^1^H-NMR) spectra were recorded at 27 °C (300 K) on an Avance III 500 MHz Bruker spectrometer equipped with a 5 mm TBI broadband probe or a 300 Ultrashield from Bruker (Billerica, MA, USA). Data were processed using the software Mestrenova (Bruker GmbH, Karlsruhe, Germany). Samples were prepared at the desired concentration in deuterium oxide (D_2_O).

### Size and zeta-potential determination

Dynamic light scattering (DLS) measurements were conducted using a Malvern ZetaSizer NanoZS with a 532 nm laser at a 173° scattering angle. PGA-Dtx solutions were filtered through a 0.45 μm cellulose membrane and measured in a DTS 1070 cell. Size distribution was assessed in triplicate at concentrations of 2, 1, 0.5, and 0.25 mg/mL in DPBS at 25 °C, with automatic beam focusing and attenuation optimization.

Zeta (ζ)-potential measurements were performed using disposable folded capillary cells, with polymer solutions prepared under the same conditions. The ζ-potential was calculated using the Smoluchowski model, with 10-30 sub-runs of 10 s each at 25 °C (n = 3).

### Circular dichroism

Circular dichroism (CD) spectroscopy was performed using a J-815 CD Spectrometer with a Peltier thermostated cell holder and a nitrogen flow controlled at 2.7 L∙min⁻¹. Samples were dissolved in DPBS and diluted to 0.5 mg/mL, then measured in a quartz cuvette (d = 0.1 cm) at 20 °C. Measurements were repeated three times (n = 3), and molar ellipticities were plotted as mean residue ellipticity.

### pH-dependent kinetics of drug release

Dtx release from PGA-Dtx was studied in PBS-based buffers at pH 7.4 (mimicking bloodstream and healthy tissues) and pH 5 (mimicking tumor and lysosomal environments). PGA-Dtx (3 mg/mL) was stirred at 37 °C, and aliquots (100 μL) were collected at fixed time points (0, 1, 2, 4, 6, 24, 48, 72 h). After each collection, samples were lyophilized, and free Dtx was extracted with methanol. The extracted samples were analyzed using liquid chromatography-mass spectrometry (LC-MS) with paclitaxel (Ptx) as an internal standard. Dtx was quantified by mass transitions specific to Dtx and Ptx in positive electrospray ionization mode. The LC-MS system used an ExionLC system with a QTRAP 4500, and the analysis followed optimized conditions: 0.5 mL/min flow rate, 70% acetonitrile in the mobile phase, and a C18 column at 40 °C.

## Results

### Multiparametric tumor growth monitoring

**Figure 1:**
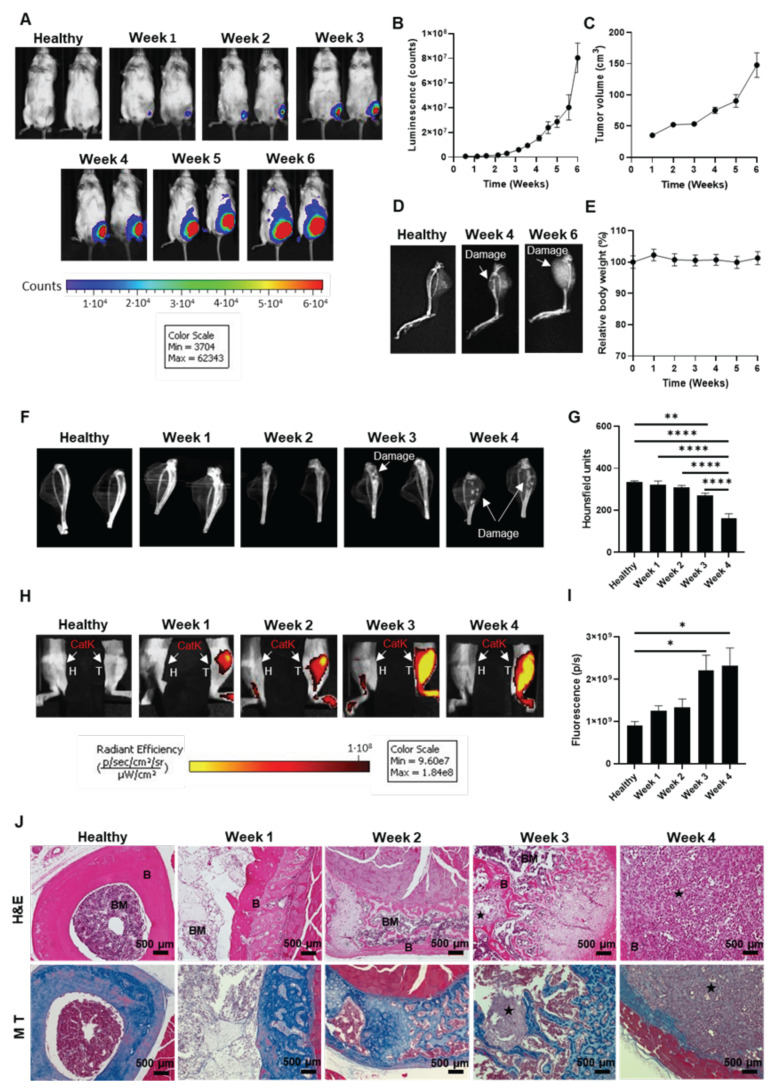
** Establishment of the Bone mCRPC Mouse Model. A**) Representative images of IVIS Spectrum luminescence data covering 6 weeks of tumor development following the injection of PC3-Luc cells into the left tibia.** B**) Graphical representation of tumor growth expressed as counts per second (n ≥ 10).** C**) Tumor volume alterations expressed in cm^3^ during tumor development (n ≥ 10). **D**) Representative IVIS Spectrum X-ray images of a healthy tibia and weeks 4 and 6 of tumor development. **E**) Graphical representation of relative body weight percentage alterations during tumor development (n ≥ 10).** F**) Representative images of micro-computed tomography (µCT) data from healthy and tumor-affected tibias during 4 weeks of tumor development. **G**) Radiodensity (expressed as Hounsfield units [HU]) of healthy and tumor-affected tibias during tumor development (n = 4).** H**) Representative IVIS Spectrum fluorescence data after administering the IVISense CatK 680 FAST probe over 4 weeks of tumor development. **I**) Cathepsin K activity expressed as photons per second from the fluorescent signal of IVISense CatK 680 FAST probe from healthy and tumor-affected tibias over four weeks of tumor development (n ≥ 3). **J**) Hematoxylin and eosin (upper images) and Masson's trichrome (lower images) staining of healthy and tumor-affected tibias over 4 weeks of tumor development. Abbreviations - B: bone; BM: bone matrix; CatK: cathepsin K; H: healthy; H&E: Hematoxylin and eosin; M T: Masson's trichrome; P/S: photons per second; star: tumor; T: tumor. Data expressed as mean ± SEM. Statistical analysis performed using ANOVA, *p < 0.05, **p < 0.01, ****p < 0.0001.

We developed the bone mCRPC mice model by the intratibial injection of androgen receptor (AR)-negative PC3 tumor cells transfected with luciferase (PC3-Luc) to support metastasis monitoring via optical imaging (**Figure S1**). After injecting PC3-Luc cells into the bone marrow cavity, we qualitatively and quantitatively measured bone growth via alterations in luminescence and bone damage via X-ray imaging to monitor tumor development over time and estimate tumor volume via tibia width and depth measurements. We observed increasing luminescence levels over 6 weeks of tumor development (**Figure 1A**); we fixed the experimental endpoint to week 6 due to ethical considerations. Quantification of luminescence revealed exponential tumor growth after 2 weeks of tumor development (**Figure 1B**). Measuring bone width/length values with a caliper also indirectly suggested an exponential increase in tumor volume after week 3 (**Figure 1C**). We observed increasing levels of bone damage over 6 weeks of tumor development in X-ray images **(Figure 1D and S2)**. Analysis of X-ray images from several tibias during tumor development failed to reveal osteolytic lesions during the first two weeks of tumor development; however, lesions appeared in 60% of mice by week 3 and all mice during weeks 4, 5, and 6 (**Figure S2** and **Figure S3A**). We then evaluated the proportion of mice with tumors within the tibia or tumors that had spread to the surrounding muscle tissue. Overall, the week 3 mice that developed tumors (60%) possessed osteolytic lesions within the tibia; however, 20% of week 4, 60% of week 5, and 100% of week 6 mice possessed tumors that had spread from the tibia into the surrounding muscle tissue (**Figure S2** and **Figure S3B**).

From a safety perspective, daily monitoring of the general condition and weight of the mice failed to reveal signs of compromised welfare over six weeks, other than mobility difficulties in week 6 due to excessive tumor growth (**Figure 1E**). Based on this observation, and to enable more precise quantification of bone damage, we adjusted the experimental endpoint to week 4, when the mice exhibited significant bone lesions in the tibia without complete fractures and maintained normal movement. At this modified experimental endpoint, we observed a significant increase in tibia volume (**Figure S4A**) and weight (**Figure S4B**) relative to total body weight in tumor-affected tibias compared to healthy tibias, supporting the presence of significant tumor growth by week 4. Importantly, we observed no significant tumor cell injection-related impact in the lungs, liver, spleen, or kidneys (**Figure S4C-G**). We did observe a significant increase in heart weight relative to total body weight in tumor-affected mice (**Figure S4G**), suggesting oxygen deficiency compensation 18.

We next evaluated and quantified bone damage in *ex vivo* samples of tumor-affected tibias over 4 weeks using micro-computed tomography (µCT) analysis. In agreement with our X-ray analysis (**Figure S2**), µCT revealed significantly increased levels of bone damage in parallel with increased tumor growth in tumor-affected tibias at weeks 3 and 4 compared to healthy tibias, described as a significant decrease in bone radiodensity (**Figure 1F and G**). Furthermore, we observed significantly increased bone damage at 4 weeks post-inoculation compared to all other time points (**Figure 1G**).

Finally, we measured and quantified lysosomal cysteine protease cathepsin K activity *in vivo*, as cathepsin K production by bone metastatic cancer cells promotes cancer cell invasion 19, and PC3-Luc cells secrete cathepsin K (**Figure S5**) and cause osteolytic lesions 20. Representative IVIS Spectrum fluorescence images during tumor development suggested a slow increase in cathepsin K activity over time (**Figure 1H**), while fluorescent signal quantification revealed a significant increase in cathepsin K activity at 3 and 4 weeks in tumor-affected tibias compared to healthy tibias (**Figure 1I**). The increase, starting at week 3, aligns with previous findings that suggest cathepsin K's involvement in osteoclast activation, a key driver of bone resorption 21, or the higher release of cathepsin K by PC3-Luc cells (**Figure S5**). The significant increases observed at weeks 3 and 4 (**Figures 1I**) coincide with the significant increases in tumor mass (**Figures 1B and C**) and bone damage levels (**Figures 1F and G**), suggesting a tight link between tumor growth and bone degradation.

### Histological analysis reveals progressive bone destruction during tumor development

After collecting macroscopic data, we aimed to evaluate evident microscopic features during tumor development via histological studies on tibia samples from previous studies. Hematoxylin and eosin (H&E)-staining of samples provided evidence for rapid tumor growth within the tibia and the progressive invasion of the bone (**Figure 1J, upper images**). Analysis at week 1 post-tumor inoculation failed to reveal bone cortical rupture or medullary involvement; however, signs of reactive cortical changes, including angiogenesis and mild chronic inflammation (mainly lymphocytes), indicated an early bone response. By week 2, angiogenesis and mild inflammation intensified as the tumor began to impact the bone environment more significantly, though the bone remained largely intact. By weeks 3 and 4, the tumor extensively invaded the bone marrow and caused a cortical rupture, extending into muscle tissue by week 4. Analysis of week 4 samples revealed increased angiogenesis, vasodilation, and chronic inflammatory infiltrates of differing degrees of lymphocytes and histiocytes and the appearance of polymorphonuclear neutrophils, indicating a more acute inflammatory response 22, suggesting a robust but ineffective immune response to the tumor. The bone and surrounding tissues became overwhelmed as the tumor continued to grow.

In Masson's trichrome-stained samples, bone (indicated by blue staining) progressively disappeared during tumor development, with virtually no bone remaining by week 4 (**Figure 1J, lower images**). Partial bone preservation during earlier stages suggested osteolytic activity but no significant loss of bone integrity. Blue-stained bone areas diminished as the tumor progressed, reflecting increasing bone degradation, which aligns with osteoclast activity typical in osteolytic bone metastasis. Analysis of week 4 revealed near-total bone loss, confirming severe osteolysis, consistent with radiological and histological findings of significant bone damage and cortical ruptures (**Figure S2** and** Figure 1F**).

### Hematological analysis reveals tumor-induced inflammation and hematopoietic disturbance

We performed hematological studies comparing mice at week 4 post-tumor inoculation with healthy mice. Tumor-bearing mice displayed increased leukocyte levels (notably neutrophils and eosinophils) (**Figure S6A-C**), a higher neutrophil-to-lymphocyte (N/L) ratio indicating poor prognosis (**Figure S6D**), but no significant changes in lymphocyte, monocyte, and band leukocyte levels (**Figure S6E-G**). Red blood cell (RBC) counts rose in tumor-bearing mice despite bone marrow disruption (**Figure S6H**), with lower mean corpuscular volume (MCV) (**Figure S6I**) and mean corpuscular hemoglobin (MCH) values (**Figure S6J**). In addition, we observed no differences in total hemoglobin concentration (**Figure S6K**) or hematocrit (**Figure S6L**), mean corpuscular hemoglobin concentration (MCHC) (**Figure S6M**), and red blood cell distribution width (RDW) (**Figure S6N**) values in tumor-bearing mice. These observations imply the possible initiation of a homeostatic response to increase RBC synthesis (typically through erythropoietin production) in response to low RBC numbers induced by the tumor insult 23. We also observed decreased reticulocyte levels (**Figure S6O**) and increased levels of immature RBC markers - the immature reticulocyte fraction (IRF, measures the number of young, undeveloped RBCs) and the highly fluorescent reticulocyte fraction (HFR; measures the number of immature young reticulocytes though high fluorescence due to high RNA content) (**Figures S6P and Q**) in tumor-bearing mice, suggesting premature RBC release. The mean platelet volume (MPV) value remained lower (**Figure S6R**), implying the presence of older platelets 24, with no change in platelet counts or plateletcrit levels (**Figure S6S and T**), indicating impaired platelet production, in tumor-bearing mice. These findings reflect a tumor-induced inflammatory response and bone marrow dysfunction.

### Analysis of the enhanced permeation and retention effect suggests an early inflammatory impact and a later increase in tumor vascularity

**Figure 2:**
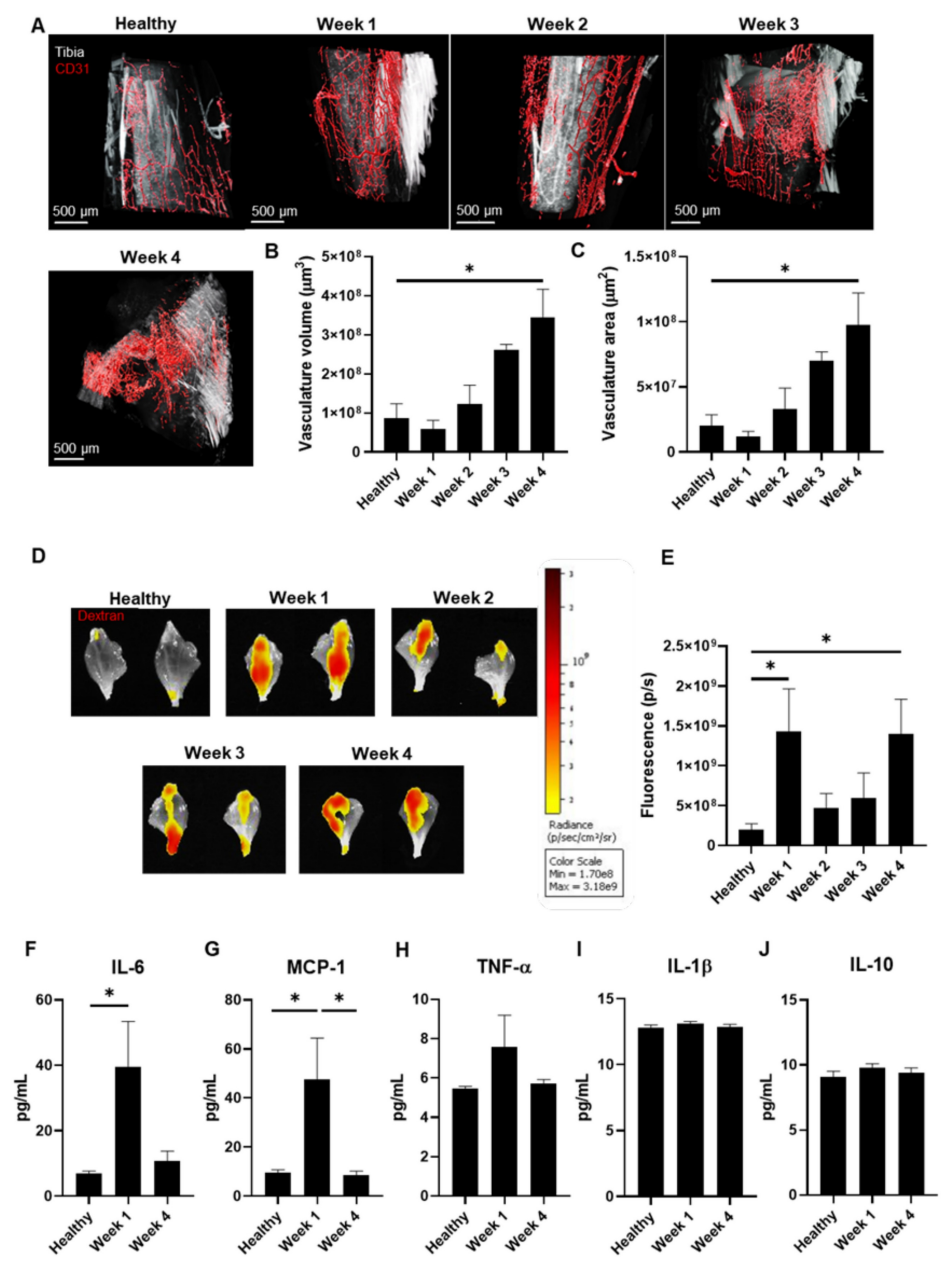
** Vasculature Status During Bone mCRPC Mouse Model Development. A**) Representative light-sheet fluorescence microscopy (LSFM) images of the tibiae-associated vasculature (CD31, in red) over 4 weeks of tumor development. Tibia signal corresponds to autofluorescence from green channel.** B and C**) Changes to the vasculature **B**) volume and **C**) area of tibiae during the 4 weeks of tumor development (represented as µm^3^ and µm^2^, respectively) (n ≥ 3). **D**) Representative IVIS Spectrum fluorescence images of mouse tibias over 4 weeks of tumor development and in healthy mice following the administration of Dextran Texas Red. **E**) Graphical representation of the relative fluorescence intensity of Dextran Texas Red in the mouse tibia over 4 weeks of tumor development and in healthy mice (n ≥ 3). **F-J**) Serum levels of **F**) IL-6, **G**) MCP-1, **H**) TNF-α, **I**) IL-1β, and **J**) IL-10 in healthy mice and tumor-affected mice at week 1 and week 4 of tumor development (n ≥ 3). Abbreviations - P/S: photons per second; pg/mL: picograms per milliliter. Data expressed as mean ± SEM. Statistical analysis performed using ANOVA, *p < 0.05.

We next employed light-sheet fluorescence microcopy (LSFM) imaging to track vascular growth with anti-CD31-AF647 antibody over four weeks of tumor development **(Figure 2A)** as described 25, 26. Analysis of healthy and week 1 tibia samples revealed a regular vascular web with long capillaries; however, shorter, irregular capillaries emerged by week 2, which became more pronounced by week 3 and widespread by week 4. Quantification of the CD31-AF647 signal confirmed increased vascular volume and surface area at week 4 compared to healthy tibias (**Figure 2B and C**). These findings indicate the induction of an angiogenic switch between weeks 2 and 3, coinciding with significantly increased tumor growth (**Figure 1B**), bone damage (**Figure 1G**), and cathepsin K activity (**Figure 1I**).

We assessed the power of the EPR effect to determine the optimal timing for nanomedicine administration 27. After injecting mice with Dextran, Texas Red™ (as a model macromolecule) weekly to assess tumor accumulation, we observed significant uptake at weeks 1 and 4 (**Figures 2D and E**) in tumor-bearing compared to healthy mice.

Given that we observed an increase in the EPR effect at week 1 when tumor vasculature remains underdeveloped and that inflammation can enhance the EPR effect 28, we evaluated inflammatory status in week 1 and 4 tumor-bearing mice (coinciding with maximal EPR) and compared data to healthy mice. We performed a multiplex assay to detect five cytokines by flow cytometry in blood serum - tumor necrosis factor-α (TNF-α), interleukin-6 (IL-6), monocyte chemoattractant protein-1 (MCP-1), and interleukin-1β (IL-1β) (all pro-inflammatory) and interleukin-10 (IL-10) (anti-inflammatory) - as previous studies had provided evidence for their implication in modulating the EPR effect 29-33. We observed significant increases in IL-6 (week 1 vs. healthy) (**Figure 2F**) and MCP-1 (week 1 vs. healthy and week 4) levels due to tumor development (**Figure 2G**), while TNF-α, IL-1β, and IL-10 levels remained similar (**Figure 2H-J**). These findings suggest that tumor-induced inflammation increases the EPR effect at week 1, whereas enhanced vascularization increases the EPR effect at week 4.

As these data revealed elevated tumor accumulation of a macromolecule via the EPR effect at week 1, we chose this time point in our subsequent studies of nanomedicine administration.

### Synthesis of a PGA-conjugated form of docetaxel

**Figure 3:**
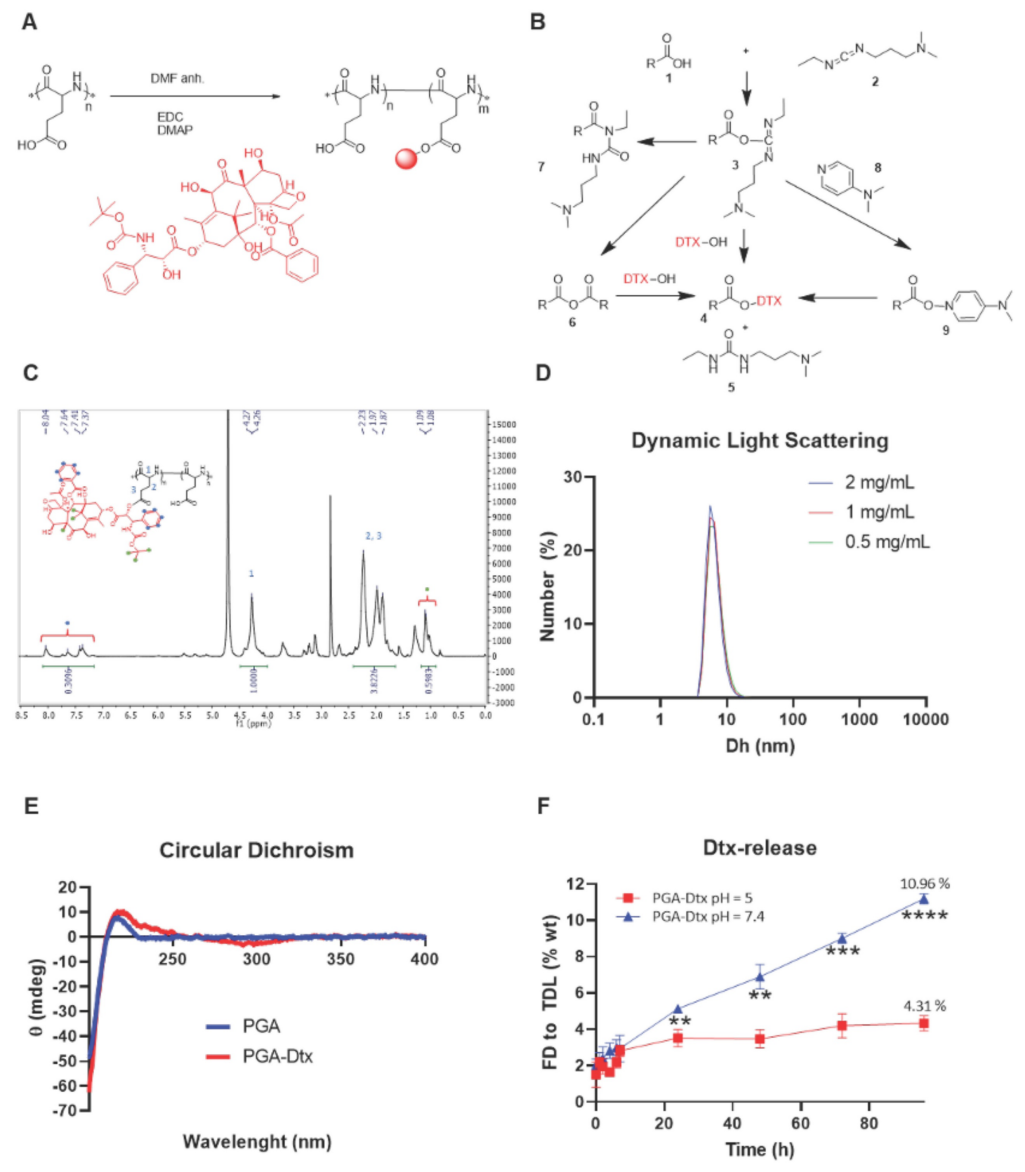
** PGA-Dtx Synthesis and Characterization. A**) Synthetic scheme for PGA-Dtx. **B**) Mechanism of EDC/DMAP ester coupling. **C**) ^1^H-NMR spectrum of PGA-Dtx. Dtx protons highlighted as green and blue circles.** D**) Size distribution by number in DPBS obtained by dynamic light scattering at 0.5, 1.0, and 2.0 mg/mL PGA-Dtx. **E**) Circular dichroism profile obtained in DPBS at 0.5 mg/mL PGA-Dtx. **F**) PGA-Dtx drug release profile by liquid chromatography-mass spectrometry at pH 5.0 and 7.4. Abbreviations - %: percentage; % wt: percentage of the total weight; Dh: hydrodynamic diameter; DMAP: 4-dimethylaminopyridine; DMF: N,N-dimethylformamide; EDC: 1-Ethyl-3-(3-dimethylaminopropyl)carbodiimide; FD: free drug; mdeg: millidegrees; mg/mL: milligram per milliliter; nm: nanometers; TDL: total drug loading; θ: molar ellipticity. Data expressed as mean ± SEM, n = 3. Statistical analysis performed using ANOVA, **p < 0.01, ***p < 0.001, ****p < 0.0001.

We conjugated Dtx to a linear PGA nanocarrier via one-step esterification in organic media using carbodiimides as crosslinkers (**Figure 3A**). The carbodiimide moieties reacted with the carboxyl groups of PGA to form an O-acylisourea intermediate (**Figure 3B**), which then reacted with the alcohol group of Dtx to yield an ester bond, forming the PGA-Dtx conjugate, and urea as a byproduct. Briefly, we dissolved linear PGA in anhydrous N,N-dimethylformamide (DMF), with 1-ethyl-3-(3-dimethylaminopropyl)carbodiimide (EDC) as a zero-length crosslinker. The water solubility of EDC and the urea byproduct allowed their straightforward removal through an aqueous media workup. The O-acylisourea intermediate could form a symmetric anhydride, react with Dtx, or rearrange into an N-acyl urea byproduct; however, a catalytic amount of 4-dimethylaminopyridine (DMAP) minimized N-acyl urea formation by stabilizing the intermediate, thus promoting the desired ester bond formation 34.

We confirmed the identity and purity of PGA-Dtx using proton nuclear magnetic resonance (¹H-NMR), detecting the aromatic protons (8.0 - 7.0 ppm) and methyl group (⁓1 ppm) signals corresponding to Dtx (**Figure 3C**). We quantified drug loading at an average value of 3.2 mol%. Finally, we fluorescently labeled PGA-Dtx sulfo-cyanine5.5-amine (Cy5.5) using 4-(4,6-dimethoxy-1,3,5-triazin-2-yl)-4-methyl-morpholinium chloride (DMTMM) chemistry, achieving 1.25 mol% Cy5.5 loading, to support subsequent assessments of *in vivo* biodistribution, tumor accumulation, and blood half-life.

### PGA-Dtx conjugate characterization

#### Drug loading, structural conformation, and zeta-potential analysis of PGA-Dtx

We quantified Dtx loading in PGA-Dtx using ultraviolet-visible (UV-VIS) absorbance at 227 nm (**Table S1**), confirming 10.08 wt % (3.33 mol %) with 0.23 wt % free Dtx, consistent with ¹H-NMR results. Dynamic light scattering (DLS) revealed a stable hydrodynamic diameter of 6.6 nm across serial dilutions (0.5, 1, and 2 mg/mL), aligning with similar PGA conjugates (**Figure 3D**) 35. Circular dichroism (CD) analysis in DPBS (0.5 mg/mL) revealed a random coil conformation, indicated by a negative band at 200 nm (**Figure 3E**). Electrophoretic light scattering (ELS) measured a zeta (ζ)-potential of -24.1 ± 2.2 mV at 1 mg/mL, suggesting enhanced tumor penetration due to reduced electrostatic retention by the extracellular matrix (ECM) 36.

#### Linker design and release kinetics of PGA-Dtx

Bone metastases exhibit microenvironmental acidosis driven by cancer cells and osteoclasts 37. Additionally, PGA-based conjugates undergo endocytic uptake and lysosomal trafficking, where acidic pH facilitates drug release 38. The pH-sensitive ester bond between PGA and Dtx ensures stability in circulation (pH 7.4) but enables targeted release through bond cleavage in acidic environments 39. We performed release studies by dissolving PGA-Dtx in relevant buffers and extracting released Dtx at selected time points. We optimized drug release studies implementing a liquid chromatography-mass spectrometry (LC-MS) methodology. We studied drug recovery from buffers used for release studies: 10 mM PB with 150 mM NaCl (PBS) at pH 7.4 or 5.0 for pH-dependent drug release (**Figure 3F**). Surprisingly, our data indicated a more significant and rapid drug release at a neutral pH of 7.4 compared to an acidic pH (pH 5); however, this data does agree with findings observed for similar systems 40.

### PGA conjugation prolongs docetaxel circulation and enhances vascular permeability and tumor accumulation

**Figure 4:**
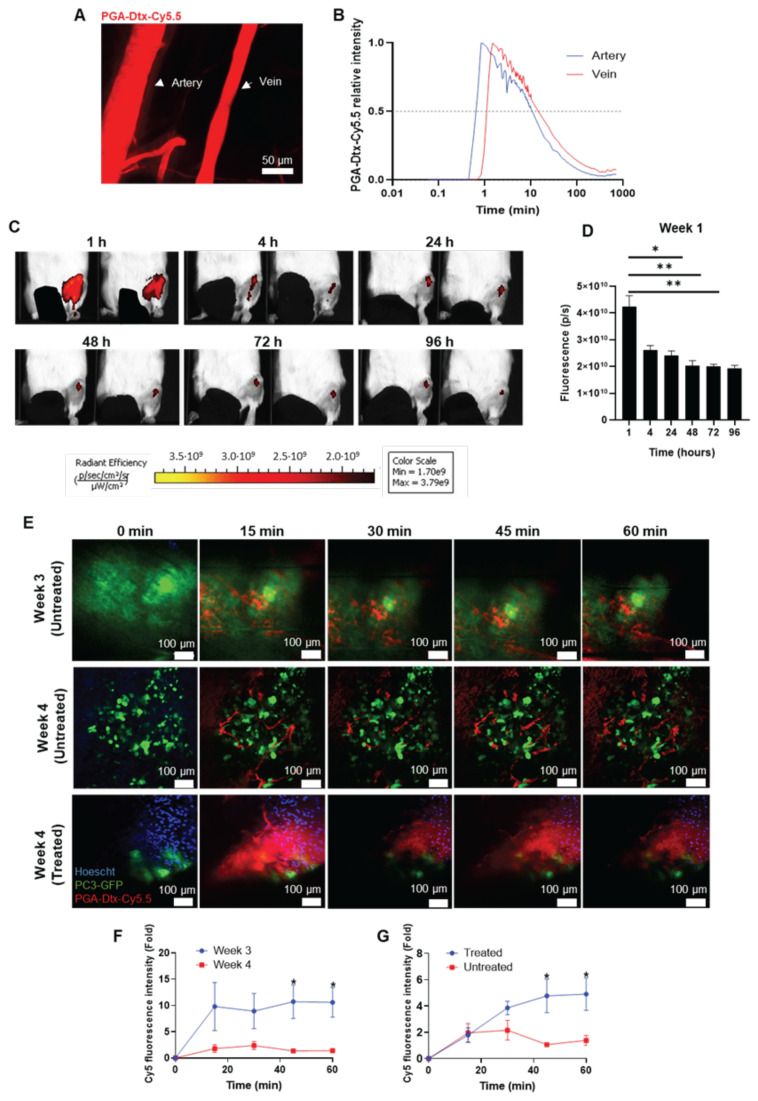
** PGA-Dtx Pharmacokinetics. A**) Representative intravital confocal microscopy (IVM) image of the mouse ear vasculature after intravenous injection of PGA-Dtx-Cy5.5. **B**) Fluorescence intensity of PGA-Dtx-Cy5.5 over time in the ear vein and artery (n = 5).** C**) Representative IVIS Spectrum fluorescence images of PGA-Dtx-Cy5.5 tumor accumulation over time (intravenous injections at week 1). **D**) Relative fluorescence intensity of PGA-Dtx-Cy5.5 over time after intravenous injection at week 1 (n = 6). **E**) Representative IVM images of PGA-Dtx-Cy5.5 diffusion into the tumor area at 0, 15, 30, 45, and 60 min after intravenous injection at week 3 (upper images) and week 4 (untreated; center images) of tumor development and at week 4 after PGA-Dtx treatment (lower images). In all cases, blue color corresponds to Hoechst signal, green color to PC3-GFP signal and red color to PGA-Dtx-Cy5.5 signal. Scale bar: 100 µm. **F**) Graphical representation of results from (**E**) comparing week 3 with week 4 (untreated) (n = 3). **G**) Graphical representation of results from (**E**) comparing week 4 (untreated) with week 4 (treated) (n ≥ 3). Abbreviations - P/S: Photons per second. Data expressed as mean ± SEM. Statistical analysis performed using ANOVA, *p < 0.05, **p < 0.01.

We evaluated conjugate stability using IVM after the intravenous injection of 7.5 mg/kg PGA-Dtx-Cy5.5; red color) (**Figure 4A**). Analysis of initial distribution half-life in circulation in healthy blood vessels revealed an average value of 17.7±4.6 min (**Figure 4B and Figure S7**) - nearly four times longer than free Dtx (4.5 min) 41. We failed to detect Cy5.5 signals outside vessels, indicating minimal extravasation into healthy tissues. This prolonged circulation time suggests enhanced tumor accumulation via the EPR effect.

We next assessed PGA-Dtx-Cy5.5 accumulation in tumor-bearing mice via IVIS after intravenous injections (7.5 mg/kg, twice weekly for six doses starting at week 1 after tumor inoculation). Monitoring PGA-Dtx-Cy5.5 tumor accumulation after the first (week 1), third (week 2), and fifth (week 3) doses at 1, 4, 24, 48, 72, and 96 h after each dose showed peak tumor accumulation at 1 h post-injection, with a gradual decline over 96 h (**Figure 4C and D; Figure S8A and B**). We also observed a significant accumulation of PGA-Dtx-Cy5.5 at 4 h for weeks 2 and 3 (**Figure S8A and B**). The highest accumulation occurred in week 1 (**Figure S8C**), with a reduced uptake in later weeks, mirroring EPR effect findings.

We also used IVM to study the tumor vasculature and PGA-Dtx-Cy5.5 accumulation in real-time over four weeks in our bone mCRPC model; in this case, we intratibially injected PC3-Luc-GFP cells during model development to support tumor cell tracking. We found tumor cell detection challenging in the first two weeks, perhaps due to insufficient tumor cell exposure to the bone surface; however, tumor cell detection improved by weeks 3 and 4 as tumors expanded from the bone marrow (**Figure 4E**). Real-time tracking revealed more significant extravasation of PGA-Dtx-Cy5.5 at week 3 (**Figure 4F**), likely due to active angiogenesis (**Figure 2A-C**). While we observed increased vasculature at week 4, we also discovered lower tumor accumulation, possibly due to restricted extravasation thanks to elevated interstitial fluid pressure (IFP) 42.

We then used IVM to compare vasculature and PGA-Dtx-Cy5.5 accumulation in treated and untreated tumor-bearing mice at the experimental endpoint. Untreated tumor-bearing mice possessed a more developed vasculature and tumor; however, PGA-Dtx-treated tumor-bearing mice lacked a defined vasculature and evident signs of tumor cells, perhaps due to diminished tumor development (**Figure 4E**). We observed poor vascularization in PGA-Dtx-treated mice but higher PGA-Dtx-Cy5.5 extravasation compared to untreated mice (**Figure 4G**), likely due to Dtx-induced vascular disruption 43.

### PGA-Dtx treatment inhibits tumor growth, reduces cathepsin K release and bone damage, and suppresses tumor-associated angiogenesis

**Figure 5:**
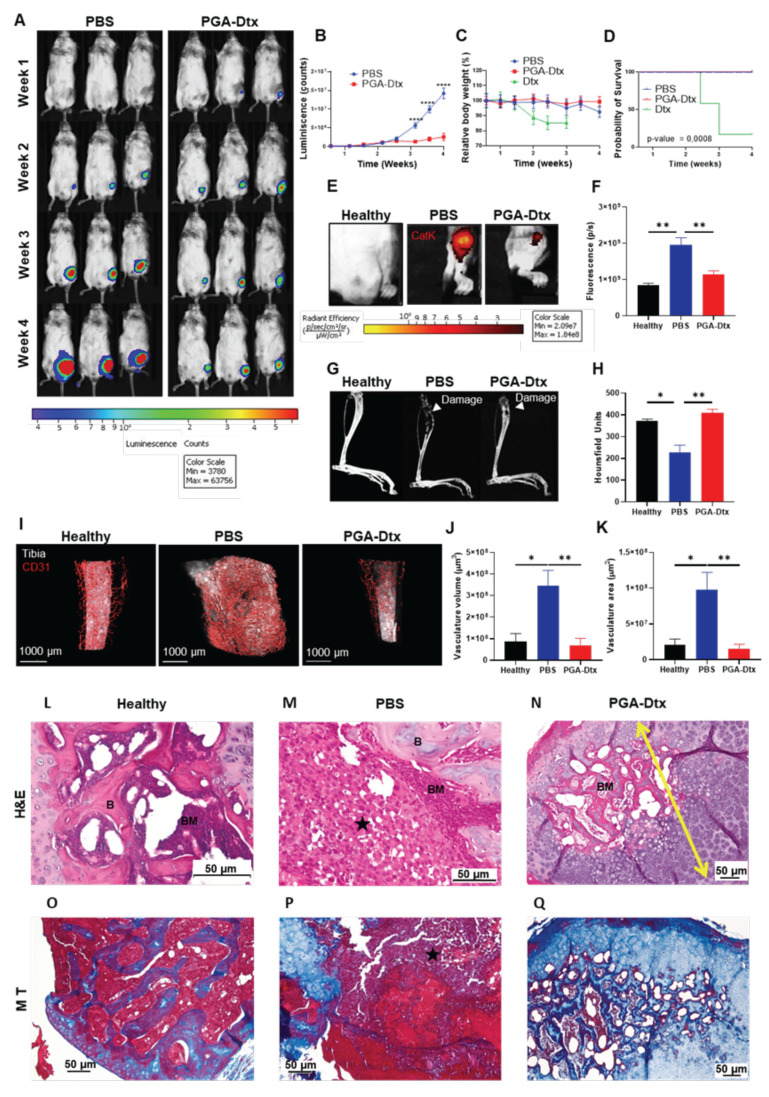
** PGA-Dtx Activity in the Bone mCRPC Mouse Model. A**) Representative images of IVIS Spectrum luminescence data over 4 weeks of tumor development in PBS- (left) and PGA-Dtx-treated mice (right) (n ≥ 8). **B**) Tumor growth expressed as counts per second in PBS- and PGA-Dtx-treated mice (n ≥ 8). **C**) Relative body weight percentage alterations for PBS-, Dtx and PGA-Dtx-treated mice over time (n ≥ 8). **D**) Kaplan-Meier survival analysis of mice treated with PBS, PGA-Dtx and free Dtx (n ≥ 8).** E**) Representative IVIS Spectrum fluorescence of the IVISense CatK 680 FAST probe at 4 weeks in healthy and PBS- and PGA-Dtx-treated mice. **F**) Graphical representation of results from (**E**) (n = 6). **G**) Representative micro-computed tomography (µCT) analysis images at 4 weeks in healthy and PBS- and PGA-Dtx-treated mice. **H**) Graphical data representation from (**G**) expressed in Hounsfield units (n ≥ 6). **I**) Representative light-sheet fluorescence microscopy (LSFM) images of the vascular system in the tibiae of healthy and PBS- and PGA-Dtx-treated mice at 4 weeks. **J and K**) Graphical representation of results from (**I**) for volume (**J**) and area (**K**) (n ≥ 3)**. L-N**) Hematoxylin and eosin staining of **L**) healthy and **M**) tumor-affected tibiae of PBS-treated and **N**) PGA-Dtx-treated mice at the experimental endpoint. **O-Q**) Masson's trichrome staining of **O**) healthy and **P**) tumor-affected tibiae of PBS-treated and **Q**) PGA-Dtx-treated mice at the experimental endpoint (n ≥ 3). Black star = tumor; yellow arrow = chondral metaplasia. Abbreviations - B: bone; BM: bone matrix; P/S: photons per second. Data expressed as mean ± SEM. Statistical analysis performed using ANOVA, *p < 0.05, **p < 0.01.

After evaluating pharmacokinetics and tumor accumulation, we studied the anti-tumorigenic activity of PGA-Dtx to validate the utility of our model. We intravenously administered treatments twice a week for three weeks, starting at week 1 of tumor development (a total of six doses). We administered PGA-Dtx and free Dtx at 7.5 mg/kg Dtx equivalent doses. Tumor growth analysis via luminescence revealed rapid tumor progression in PBS-treated mice, while PGA-Dtx significantly suppressed tumor growth, reducing tumor expansion by 81.7% (**Figure 5A and B**). We could not collect luminescence data from free Dtx group due to poor survival of these mice. In contrast to free Dtx, analyses in PGA-Dtx-treated mice revealed a lack of toxicity, demonstrating the benefits of PGA conjugation (**Figure 5C, D and S9**). The elevated level of toxicity of free Dtx following this schedule highlights its narrow therapeutic window 44. We confirmed reduced tumor burden in PGA-Dtx-treated mice, with tibia weight and volume measurements similar to healthy controls (**Figure S10A** and **B**). Significant alterations included increased heart weight in PBS-treated mice (**Figure S10C**) and increased spleen weight in PGA-Dtx-treated mice (**Figure S10D**); however, we observed no significant differences in lung, liver, or kidney weights (**Figures S10E-G**). The increased spleen weight observed after PGA-Dtx treatment related to the effect of Dtx rather than PGA (**Figure S11**), demonstrating the efficient delivery of Dtx as part of the PGA-Dtx nanoconjugate, which likely drives immunogenic cell death.

Further analyses revealed significantly increased cathepsin K activity in PBS-treated mice compared to healthy tibias; however, cathepsin K activity levels remained similar between PGA-Dtx-treated and healthy mice, indicating that PGA-Dtx reduced the release of cathepsin K from PC3 cells/osteoclasts in tumor-bearing mice (**Figure 5e** and** F**). To note, as cathepsin K is a well-established downstream effector of RANKL-mediated osteoclast activation, its expression serves as a functional readout of RANKL/OPG signaling 19-21. Therefore, by assessing cathepsin K activity, we indirectly evaluate the impact of the RANKL/OPG pathway on osteoclast-mediated bone remodeling. µCT analysis revealed significant bone damage in PBS-treated mice, whereas PGA-Dtx treatment preserved bone structure, maintaining bone integrity comparable to healthy tibias (**Figure 5G and H**). These findings suggest that PGA-Dtx treatment can prevent tumor-induced bone degradation maintaining bone integrity.

We also evaluated the effects of PGA-Dtx treatment on the vascular component of the TME by studying vascular status with LSFM at the experimental endpoint. PBS-treated mice displayed increased tumor-associated vasculature with abnormal, irregular capillaries, typical of tumor growth 45. In contrast, PGA-Dtx-treated mice displayed vasculature similar to healthy tibias, with an irregular but less-developed pattern (**Figure 5I**). Apart from tumor growth inhibition by PGA-Dtx treatment and the related lack of an increased vascular network, the previously explained effects of Dtx on the tumor vasculature could explain the irregular pattern of the existing vasculature 43. Quantification of LSFM images confirmed significantly lower tumor vasculature volume (**Figure 5J**) and area (**Figure 5K**) in PGA-Dtx-treated mice compared to PBS-treated mice, with PGA-Dtx-treated mice displaying values similar to the healthy tibia.

### Tibia histology reveals bone marrow and bone structure restoration after PGA-Dtx treatment

H&E staining of healthy tibias revealed signs of normal bone, with cortical and trabecular bone, articular cartilage, and growth cartilage, separating epiphysis from diaphysis, while the bone marrow presented abundant hematopoietic tissue (90-100%) and few (<10%) adipocytes (**Figure 5L and S12A**). Analysis of tibial sections from PBS-treated mice demonstrated the almost total occupation of the medullary space of the diaphysis and, in some cases, of the epiphysis by tumor tissue with little residual hematopoietic tissue (**Figure 5M and S12B**). The tumor also caused the rupture of the bone cortex, with extension to the surrounding muscle (**Figure 5M and S12B**). We failed to observe any tumor cellularity in tibial sections of PGA-Dtx-treated mice (**Figure 5N and S12C**). However, we did detect involutional fibrous tissue in all cases, suggesting that the tumor had been replaced by a reactive scar process with chondral metaplasia and osteogenesis in distinct stages. In fact, hematopoietic tissue formation began to be seen in the space previously occupied by the tumor, occupying around an 80% of the space, with the remainder occupied by adipose tissue.

Masson's trichrome staining of healthy tibias revealed cortical bone at the fragment edge (intense blue color) and trabecular bone (small blue trabeculae) between the hematopoietic tissue (**Figure 5O and S12D**). Masson's trichrome staining highlighted the tumor, which occupied the entire medullary space, with the disappearance of the bone trabeculae in PBS-treated mice (**Figure 5P and S12E**) but revealed osteochondral tissue, replacing the space previously occupied by the tumor and the formation of hematopoietic tissue in PGA-Dtx-treated mice (**Figure 5Q and S12F**). These results indicate that PGA-Dtx inhibits tumor growth and helps maintain bone structure and marrow composition, which has significant therapeutic and clinical implications.

### Spleen histology reveals a balanced immune response after PGA-Dtx treatment

The spleen of healthy mice revealed a typical pattern, with poorly defined white pulp and a red pulp composed of a network of sinusoids rich in red blood cells, together with multinucleated giant cells and the formation of small periarteriolar lymphoid nodules (**Figure S12G**). Histological sections of the spleen in PBS-treated mice revealed a reactive pattern, with periarteriolar hyperplasia of the white pulp, forming larger nodules than those of healthy mice, along with a slight increase in the lymphocyte population in the red pulp and a decrease in multinucleated giant cells (**Figure S12H**). Histological sections of PGA-Dtx-treated mice spleens revealed a pattern similar to healthy mice; compared to PBS-treated mice, nodular hyperplasia of the white pulp disappeared, but, unlike healthy mice, an increase in lymphoid tissue in the red pulp remained (**Figure S12I**). PGA-Dtx treatment partially restored spleen homeostasis, reversing periarteriolar hyperplasia observed in PBS-treated mice while maintaining increased lymphoid tissue in the red pulp, suggesting an ongoing and more controlled immune response.

### PGA-Dtx treatment restores hematopoiesis, induces lymphocyte-based response, and triggers pro-inflammatory cytokine release

Blood sample analysis revealed that PGA-Dtx treatment increased RBC count and hemoglobin levels while reducing the MCV value. PGA-Dtx treatment also increased the RDW value, suggesting the production of new, larger RBCs which coexist with older, smaller cells, indicating bone marrow repair and enhanced RBC production (**Figure S13A-F**). Additionally, PGA-Dtx treatment restored reticulocyte levels to healthy values, improved immature reticulocyte maturation (**Figure S13G-I**), and enhanced oxygen-carrying capacity (as shown by the recovery of heart weight; **Figure S10C**). Platelet count decreased, but the MPV value increased, indicating the destruction of older platelets and the production of larger, younger platelets 50, potentially improving treatment outcomes (**Figure S13K-M**) 46. PGA-Dtx treatment also increased leukocyte numbers (lymphocytes, in particular) without exacerbating neutrophil or eosinophil counts, suggesting better inflammatory control and slower disease progression (**Figure S13N-T**).

Finally, an analysis of serum levels of cytokines in healthy and PBS- and PGA-Dtx-treated mice failed to reveal any significant differences in any of the cytokines evaluated between healthy and PBS-treated mice (**Figure S14A-E**); however, we did observe significantly higher levels of TNF-α (**Figure S14A**), IL-6 (**Figure S14B**), and MCP-1 (**Figure S14C**) but not IL 1-β (**Figure S14D**) and IL-10 (**Figure S14E**) in PGA-Dtx-treated mice compared to healthy and PBS-treated mice. These data align with the increase of a lymphocyte-mediated response derived from PGA-Dtx treatment, leading to this pro-inflammatory cytokine secretion and providing an anti-tumor effect.

## Discussion

We pursued the development and exhaustive characterization of an intratibial bone mCRPC mouse model that recapitulates the complexity of the disease and responds to the specific needs of nanomedicine preclinical evaluations, as supported by our analysis of PGA-Dtx. We chose to analyze PGA-Dtx in this model based on the current clinical PCa treatments and the clinical benefits reported for PGA-based conjugates 12-15.

Unlike previous models focusing solely on osteoblastic or osteolytic features 47, our optimized model provides a comprehensive framework by integrating tumor progression, osteolysis, angiogenesis, and systemic effects. Our model effectively demonstrated tumor-induced osteolytic lesions, highlighting cathepsin K as a marker and driver of bone destruction. While cathepsin K inhibitors have been explored for osteoporosis 48, their potential in cancer-related bone loss remains under-evaluated. We propose the development of novel approaches to PCa treatment that exploits cathepsin K's enzymatic activity for targeted drug delivery; for example, the design of cathepsin K-responsive drug carriers with selectively cleavable linkers (e.g., Gly-Lys) may enhance therapeutic specificity 49. This strategy offers a promising avenue to minimize systemic toxicity while maximizing drug efficacy in the tumor-bone microenvironment.

H&E staining revealed a clear timeline of tumor progression in our model, from early reactive changes in the bone cortex to cortical rupture and tumor invasion into surrounding tissues. The increasing level of angiogenesis, inflammatory infiltration, and rapid tumor expansion underscored the model's aggressive nature and impact on the bone microenvironment. These findings aligned with radiological and fluorescent imaging results, confirming significant histological changes accompanying tumor invasion. Masson's trichrome staining further corroborated the osteolytic nature of our model, emphasizing its value in assessing bone degradation. The observed cortical damage, angiogenesis, and inflammation highlight key processes that, if disrupted, could slow tumor growth and bone destruction.

Hematological studies in tumor-inoculated mice revealed significant immune and erythropoietic disruption, underscoring the systemic impact of bone metastatic PCa 76. Elevated levels of leukocytes (particularly neutrophils and eosinophils) correlated with an increased N/L ratio and enhanced tumor-associated inflammation, which aligned with histological findings of neutrophil infiltration 50. Additionally, tumor progression impaired erythropoiesis, producing smaller RBCs with lower hemoglobin content, which reduced oxygen transport efficiency and contributed to cardiac hypertrophy as a compensatory response 51. We also observed disrupted platelet production, with lower MPV values indicating a bigger population of older platelets due to the inability of producing new platelets 24. While N/L ratio elevation represents a known prognostic marker in advanced cancer 50, our findings extend prior research by linking hematopoietic dysfunction to systemic consequences such as cardiac strain. These disruptions, potentially driven by tumor-induced bone marrow suppression and inflammation, highlight therapeutic opportunities in targeting erythropoietin-related pathways or megakaryocyte function; however, the precise mechanisms involved and the potential for reversibility of hematological changes remain unclear, warranting further longitudinal studies to determine their relationship to tumor progression, systemic inflammation, and bone marrow infiltration.

LSFM studies revealed increased vascular volume and area alongside tumor progression, highlighting angiogenesis as a key mechanism supporting tumor growth by enhancing nutrient and waste exchange 52. The presence of numerous short capillaries expanded the diffusion surface, facilitating efficient exchange processes 53. Additionally, the increased EPR effect observed at week 4 correlates with increased angiogenesis (promoting greater blood vessel density and permeability within tumors), which facilitates the accumulation and retention of macromolecular drugs/nanomedicines at the tumor site. Their structurally abnormality (dense, tortuous, and often lacking proper basement membranes and surrounding smooth muscle cells) makes these vessels significantly more permeable than normal tissue vasculature 54, 55. Histological and imaging analyses further mapped tumor progression from cortical changes to angiogenesis, reinforcing the therapeutic potential of anti-angiogenic strategies, such as tyrosine kinase inhibitors (e.g., axitinib, cabozantinib) 56. Biodistribution studies revealed higher dextran accumulation at week 1, likely due to early inflammation-driven permeability, and at week 4, attributed to increased vascularization. Elevated IL-6 and MCP-1 levels at week 1 suggested their role in sustaining inflammation, increasing VEGF production, and enhancing vascular permeability, which supports nanoparticle accumulation 29. These findings highlight the dynamic interplay between inflammation, angiogenesis, and drug delivery in bone metastases, suggesting that timing nanoparticle-based therapies to specific tumor stages could optimize treatment efficacy. However, the pharmacological properties of PGA-Dtx strengthen therapeutic performance across different tumor stages. The design of PGA-Dtx ensures extended circulation time, high stability, and a sustained release profile, which enhance drug availability to tumors independent of the implication of the EPR effect. Additionally, PGA-Dtx´s impact on the vascular component of the TME (to increase PGA-Dtx accumulation at the tumor site) and the potential role of tumor-associated macrophages (uptake administered nanomedicines and then serve as depots for their gradual release and exposure to neighboring tumor cells) also influence PGA-Dtx accumulation independent of the EPR effect 57. These characteristics may help mitigate patient-to-patient variability in the EPR effect and provide therapeutic benefit even when passive accumulation is not at its peak. Future research should explore how modulating inflammatory and angiogenic pathways may improve nanoparticle retention and penetration while also assessing long-term outcomes, such as tumor recurrence and post-treatment bone repair.

PGA-Dtx characterization demonstrated consistent drug loading (3.33 mol %), stability, a favorable size (6.6 nm), and a negative zeta potential (-24.1 mV), supporting effective tumor penetration. Release studies demonstrated higher Dtx release at neutral pH driven by structural transitions in PGA, which agrees with findings in similar systems 40. At pH 7.4, PGA adopts an open random coil formation that increases linker accessibility and drug release; meanwhile, protonation induces a rigid alpha-helix at pH 5.5, thereby limiting release 40. These findings highlight the potential for optimizing PGA-Dtx conjugates to enhance pH-responsive drug release. Future strategies may refine the PGA backbone (e.g., hydrophobic side chains for stability at neutral pH but destabilization at acidic pH) 58 or modify linker chemistry (e.g., hydrazone, imine, or acetal cross-linking) 59 to improve release within the acidic bone TME, enhancing therapeutic efficacy. Importantly, while we understand the complex nature of the *in vivo* TME, we only considered pH when carrying out the release study. Enzymes abundantly expressed in tumor tissues (e.g., cathepsin B) facilitate PGA-Dtx cleavage to accelerate Dtx release 12, 60-62. These enzymatic and additional microenvironmental cues, absent in our simplified pH-based *in vitro* release study, has been demonstrated in our previous work 61 and in clinical-stage PGA conjugates such as Opaxio, which achieved complete tumor regression in preclinical models despite minimal free drug detected early post-injection 12-15. These combined microenvironmental triggers likely contribute to the therapeutic efficacy observed *in vivo*, compensating for the slower release observed at acidic pH *in vitro*.

Intravenous PGA-Dtx injection supported extended circulation times, increasing the initial distribution half-life of Dtx by nearly fourfold and enhancing tumor accumulation via the EPR effect, which agrees with findings for other PGA-conjugates 12, 15, 61-63. Despite increased vascularity at week 4, reduced tumor accumulation may stem from elevated interstitial fluid pressure (IFP), which compresses vessels and hinders nanoparticle extravasation 42. In contrast, PGA-Dtx-treated mice exhibited minimal tumor vasculature and tumor signal, suggesting inhibited tumor growth and improved drug penetration due to reduced IFP and Dtx-induced vascular permeability 43. Future optimization should focus on exploring and overcoming IFP-related barriers by combining PGA-Dtx with ECM-modifying agents (e.g., lipoxygenase inhibitors) 63 or IFP-lowering strategies (e.g., erlotinib, mild hyperthermia) 64. Next-generation PGA-Dtx nanoconjugates incorporating tumor-targeting ligands (e.g., Ephrin type-A receptor 2, bisphosphonates) may also enhance specificity and/or reduce off-target clearance 65, 66. Future studies should assess precise quantification of tumor payload delivery through phantom-based fluorescence calibration with *ex vivo* LC-MS (%ID/g) or mass spectrometry imaging to map and quantify intratumoral distributions of PGA-Dtx and released Dtx 67, 68. These approaches would enable direct comparison of payload delivery between PGA-Dtx and free Dtx beyond the semi-quantitative fluorescence data reported here.

PGA-Dtx treatment significantly reduced tumor growth, restored normal heart weight, hematopoietic capacity, and oxygen diffusion, and mitigated tumor-induced bone damage due to impeded tumor growth; meanwhile, free Dtx treatment was not well-tolerated, and treated animal did not reach the experimental endpoint due to toxicity-related issues, which highlights the advantages of PGA conjugation in mitigating side effects. The reduced systemic side effects of PGA-Dtx arise from pharmacokinetic and biodistribution control. PGA-Dtx displayed limited extravasation in healthy vessels, prolonged circulation (≈4× longer half-life vs. free Dtx), and improved tolerability while maintaining robust anti-tumor efficacy (≈81.7% tumor growth inhibition vs. PBS). Moreover, the avoidance of commonly employed excipients in clinical Dtx formulations (e.g., Tween-80 and ethanol), which can cause hypersensitivity reactions and other unwanted side effects, further contributes to a favorable safety profile 69. The observed increase in spleen weight suggests an immune response (possibly via enhanced leukocyte production), as previously observed for PGA-drug conjugates, and erythrocyte homeostasis 70-72. PGA alone did not cause an increase in spleen weight, consistent with previous reports demonstrating that PGA-based carriers are non-immunogenic and do not induce splenomegaly 73. Although we carried out spleen measurements in the free Dtx group at a different time point due to early mortality, we observed a similar increase, which indicated a link between spleen enlargement and the immunostimulatory effects of Dtx after delivery instead of the effect of the carrier alone 74-76. Evaluating the activation/recruitment of immune cells (e.g., T cells and macrophages) may identify pathways through which PGA-Dtx enhances anti-tumor immunity. Future formulations may incorporate immune-stimulating adjuvants to amplify therapeutic effects. Additionally, PGA-Dtx's ability to mitigate tumor-induced bone damage opens avenues for its application in other bone-metastatic cancers. Combining PGA-Dtx with agents that promote bone regeneration or prevent osteolytic activity (e.g., bisphosphonates or receptor activator of nuclear factor kappa-β ligand (RANKL) inhibitors 66, 76) may enhance bone preservation and overall patient outcomes. PGA-Dtx also suppressed cathepsin K activity, reducing osteolytic effects and limiting tumor expansion, highlighting its potential for bone-metastatic cancers (e.g., breast cancer and multiple myeloma) 77, 78. The ability to monitor cathepsin K activity may also help track tumor evolution. µCT findings suggested that PGA-Dtx-treated mice did not develop bone-damaging tumors, halting bone damage and allowing endogenous bone repair. We hypothesize that the inhibition of cathepsin K release/activity associated with PGA-Dtx-mediated tumor growth inhibition prevents excessive bone resorption, thus reducing bone damage and limiting tumor invasion and metastasis as the tumor has no space to expand. Additionally, treatment reduced tumor vascular volume and area, suggesting effective angiogenesis inhibition, which may restrict metastasis 79. TME alterations may also have broader implications for other components (e.g., immune cell infiltration and tumor hypoxia) influenced by vascular changes 80. Future studies may investigate how PGA-Dtx impairs angiogenesis, potentially exploring its interactions with key signaling pathways (e.g., VEGF or hypoxia-inducible factors) that govern tumor growth. The impact of PGA-Dtx on tumor oxygenation and its subsequent influence on gene expression, immune cell behavior, and tumor progression may reveal novel approaches for enhancing the therapeutic efficacy of PGA-Dtx. Expanding the use of PGA-Dtx to other cancer types with known angiogenic dependencies (e.g., glioblastoma or ovarian cancer) may determine its broader applicability 81, 82.

Histological analysis revealed that untreated tumor-bearing mice exhibited severe bone degradation and marrow displacement, consistent with the model's osteolytic nature. In contrast, PGA-Dtx-treated mice lacked tumor cells in the bone, indicating significant tumor elimination. Chondral metaplasia, osteogenesis, and increased marrow adiposity suggested active bone repair and remodeling after tumor eradication, potentially restoring bone integrity and reducing complications like pain and fractures 83. Additionally, the absence of tumor cells in the bone marrow implied hematopoietic recovery, which is crucial for systemic health and immune resilience 84. Spleen analysis revealed that PGA-Dtx-treated mice exhibited balanced immune function, resolving tumor-induced inflammation while maintaining immune surveillance. These findings suggest that PGA-Dtx promotes bone regeneration and restores immune homeostasis by inhibiting tumor growth, reducing the risk of chronic inflammation. Future studies should explore the molecular mechanisms underlying bone repair and immune modulation to enhance its therapeutic potential in bone-metastatic cancers.

Hematological findings suggest that PGA-Dtx treatment enhanced RBC production and quality by restoring hemoglobin levels and reticulocyte maturation while preventing bone marrow damage. Unlike the compensatory RBC increase observed in untreated tumor-bearing mice, which results from a compensatory mechanism that attempts to deliver oxygen to the body more efficiently in a bone marrow-deficient situation 18, 51, PGA-Dtx-driven RBC production correlates with normalized heart weight, indicating improved oxygen delivery. This fact suggests that the increased RBC production in PGA-Dtx-treated mice is dissimilar to the increased RBC production in the control group, as cardiomegaly becomes reversed in PGA-Dtx-treated mice. Despite a decrease in platelet count and plateletcrit, MPV normalization suggests ongoing platelet production, indicative of bone marrow recovery; moreover, lower platelet numbers may contribute to improved therapeutic outcomes, reducing tumor growth and resistance to therapy 46. Increased leukocyte and lymphocyte counts, splenomegaly, and enhanced lymphoid tissue indicate activated lymphocyte-mediated anti-tumor immunity derived from PGA-Dtx treatment, as observed for other PGA-drug conjugates 70, 71. Elevated TNF-α, IL-6, and MCP-1 levels further support immune activation, suggesting that PGA-Dtx triggers a lymphocyte-mediated immune response that may contribute to anti-tumor effects 85, while unchanged IL-1β and IL-10 levels suggest that this response remains specific to certain inflammatory pathways and does not become counterbalanced by anti-inflammatory signals. Analysis of hematopoietic tissue in tibias from PGA-Dtx-treated mice suggests spleen involvement in post-treatment blood cell regeneration, leading to enlargement due to extracellular hematopoiesis 86. Future research should explore how PGA-Dtx modulates immune responses, regulates cytokine activity, and interacts with the inflammatory microenvironment, potentially informing combination therapies with immunotherapies. Understanding the role of PGA-Dtx in extramedullary hematopoiesis may also improve strategies for restoring hematopoietic function and long-term immune resilience in cancer patients. Further research may also investigate whether similar benefits occur in alternative cancer types, providing a broader therapeutic scope for PGA-Dtx beyond PCa.

The novelty of this study lies not in the development of a single material, but in the integration of three complementary components that together address long-standing barriers in nanomedicine translation: (i) the optimization and exhaustive multiparametric characterization of an intratibial mCRPC model, establishing a reproducible preclinical platform; (ii) the synthesis of a novel PGA-Dtx conjugate, distinct from previously reported PGA-taxane systems; and (iii) the application of this optimized model to evaluate nanomedicine efficacy in the clinically relevant setting of bone mCRPC. This systems-level integration represents a translational bridge that aligns with recent strategic priorities in the field 87-90 and provides a robust platform for evaluating nanotherapeutics in relevant pathophysiological settings.

## Conclusions

This study successfully developed and characterized an intratibial bone mCRPC mouse model designed as a platform for nanomedicine evaluation. Intratibial injection of PC3-Luc cells in immunodeficient mice led to predictable tumor growth, progressive bone damage, and systemic inflammation, with imaging and histological analyses confirming tumor invasion, angiogenesis, and aberrant vasculature development. We also synthesized and characterized a PGA-Dtx conjugate with optimal physicochemical properties, sustained drug release, and effective tumor accumulation. PGA-Dtx treatment significantly delayed tumor growth, reduced cathepsin K activity, preserved bone integrity, prevented anemia, and induced an inflammatory response suggestive of enhanced immune activation. While showing great promise, this study employed PGA-Dtx as a model nanomedicine and additional effort will be required to advance this therapeutic approach nearer to clinical evaluation. As such, the identification and development of novel therapeutic approaches for mCRPC patients lie beyond the scope of this study. These findings demonstrate PGA-Dtx's potential as a therapeutic strategy for bone-metastatic PCa and highlight our model's utility in evaluating novel nanomedicine treatment strategies that target bone metastases.

## Supplementary Material

Supplementary figures.

## Abbreviations

% mol: mole percent
% wt: percent weight
µCT: micro-computed tomography
^1^H-NMR: proton nuclear magnetic resonance
3D: three-dimensional
ABC: adenosine triphosphate-binding cassette
Abz-HPG⁓GPQ-EDN₂ph: anthranilamide-HPG⁓GPQ-2,4-dinitrophenil
ANOVA: analysis of variance
AR: androgen receptor
ATP: adenosine triphosphate
bFGF: basic fibroblast growth factor
CAFs: cancer-associated fibroblasts
CA-IX: carbonic anhydrase IX
Cat K: cathepsin K
CD: circular dichroism
CD31: cluster of differentiation 31
CD31-AF647: cluster of differentiation 31-alexa fluor 647
CRPC: castration-resistant prostate cancer
Cy5.5: sulfo-cyanine5.5
D_2_O: deuterium dioxide
ddH_2_O: double distilled water
Dh: hydrodynamic diameter
diDHA: di-docosahexaenoic acid
DIEA: N,N-diisopropylethylamine
DLS: dynamic light scattering
DMAP: 4-dimethyl aminopyridine
DMF: N,N-dimethylformamide
DMTMM BF4: 4-(4,6-dimethoxy-1,3,5-triazin-2-yl)-4-methyl-morpholinium tetrafluoroborate
DPBS: Dulbecco's phosphate-buffered saline
DTT: dithiothreitol
Dtx: docetaxel
ECM: extracellular matrix
EDC: 1-ethyl-3-(3-dimethylaminopropyl)carbodiimide
EDTA: ethylenediaminetetraacetic acid
ELS: electrophoretic light scattering
EPR: enhanced permeability and retention
FA: formic acid
FBS: fetal bovine serum
FD: free drug
FOV: field of view
G418: geneticin
GAU: glutamic acid units
GFP: green fluorescent protein
H&E: hematoxylin and eosin
HBSS: Hank's Balanced Salt Solution 1X
HFR: highly fluorescent reticulocyte fraction
HOBt: 1-hydroxibenzotriazol monohydrate
HU: Hounsfield units
IFP: interstitial fluid pressure
IL-10: interleukin 10
IL-1β: interleukin-1β
IL-6: interleukin 6
Inhb: inhibitor
IRF: immature reticulocyte fraction
IVM: intra-vital microscopy
LC-MS: liquid chromatography-mass spectrometry
LOD: limit of detection
LOQ: limit of quantification
LSFM: light-sheet fluorescent microscopy
Luc: luciferase
MCH: mean corpuscular hemoglobin
MCHC: mean corpuscular hemoglobin concentration
MCP-1: monocyte chemoattractant protein-1
mCRPC: metastatic castration-resistant prostate cancer
MCV: mean corpuscular volume
MDR: multidrug resistance
MPV: mean platelet volume
MW: molecular weight
MWCO: molecular weight cut-off
N/L: neutrophil-to-lymphocyte ratio
NHE-1: sodium-hydrogen exchanger-1
PBS: phosphate-buffered saline
PCa: prostate cancer
PE: phycoerythrin
PFA: paraformaldehyde
PGA: poly-L-glutamic acid
PGA-Dtx: poly-L-glutamic-docetaxel
PGA-Dtx-Cy5.5: poly-L-glutamic-docetaxel-sulfo-cyanine5.5
pRNA: plasmid RNA
Ptx: paclitaxel
RBC: red blood cells
RDW: red blood cell distribution width
RLU: relative luminescence units
RNA: ribonucleic acid
ROI: region of interest
RPMI-1640: Roswell Park Memorial Institute-1640
SAPE: streptavidin-R-phycoerythrin
SEM: standard error of the mean
TDL: total drug loading
TME: tumor microenvironment
TNF-α: tumor necrosis factor-α
UV-VIS: ultraviolet-visible spectrometry
V-ATPase: vacuolar-H+-ATPase
VEGF: vascular endothelial growth factor
λem: emission wavelength
λex: excitation wavelength
