# Supplementary material for: Characterization and validation of a bone metastatic castration-resistant prostate cancer model as a nanomedicine evaluation platform
Source: Theranostics. 2026 Jan 1;16(7):3556–76. doi: 10.7150/thno.123005 (PMC12846746; doi:10.7150/thno.123005)
Supplement: Supplementary file 1 — Supplementary figures. [file thnov16p3556s1.pdf]

Supplementary Figures

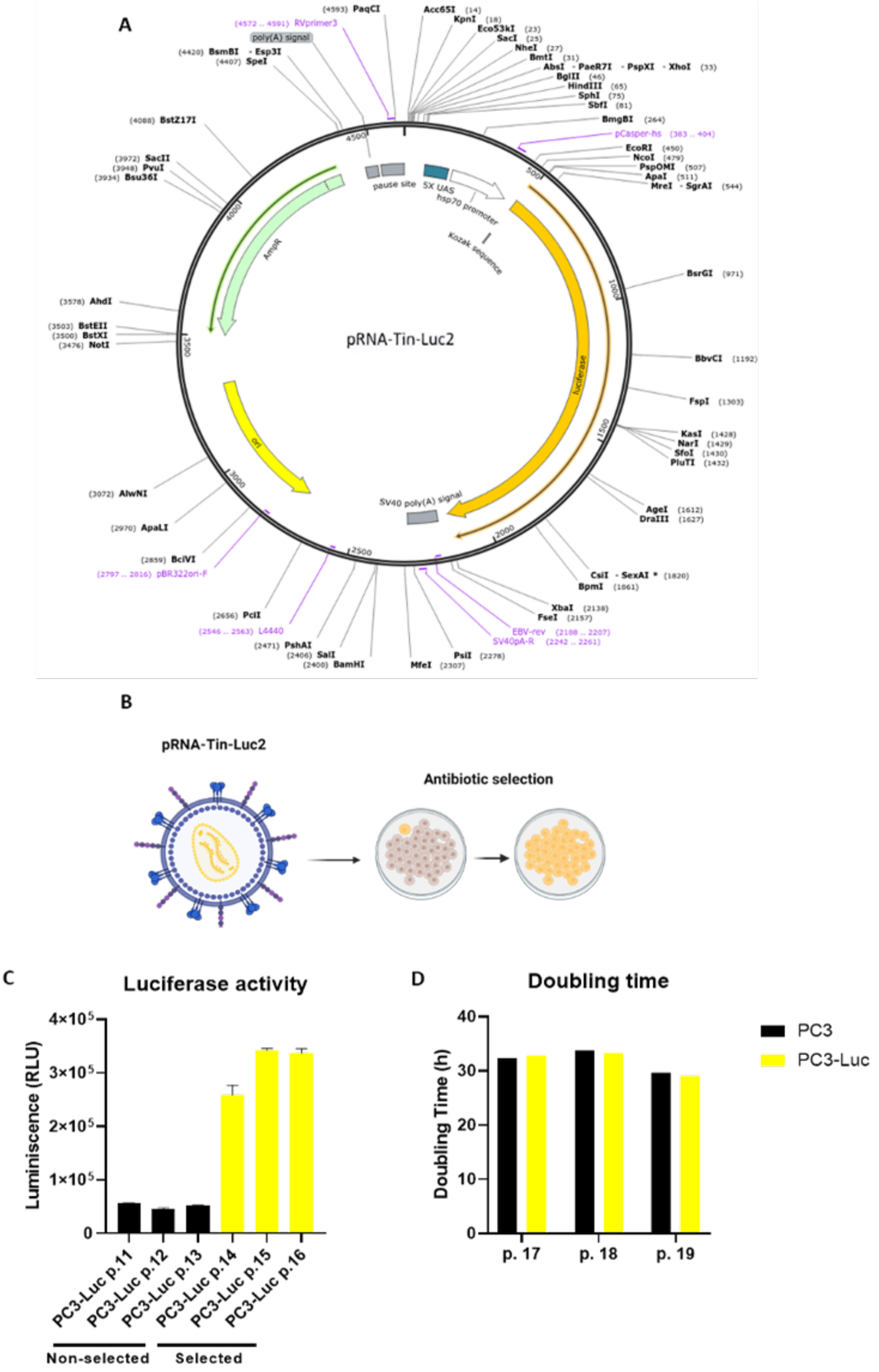

Figure S1. Lentiviral Infection of PC3 Prostate Cancer Cells with the Luciferin Transgene. A) The pRNATin-Luc2 plasmid expresses the Luciferase transgene under the control of the T3 promoter and a G418 (geneticin) antibiotic resistance cassette. **B**) PC3 cells were infected with lentiviral particles containing the pRNATin-Luc2 plasmid, and luciferase-positive cells (PC3-Luc cells) were selected using G418. **C**) Luminescence analysis of luciferase expression demonstrates efficient and stable transfection. **D**) Doubling time of PC3 and PC3-Luc cell lines for three different

passages. Data expressed as mean ± SEM (n = 3). Abbreviations- RLU: relative luminescence units.

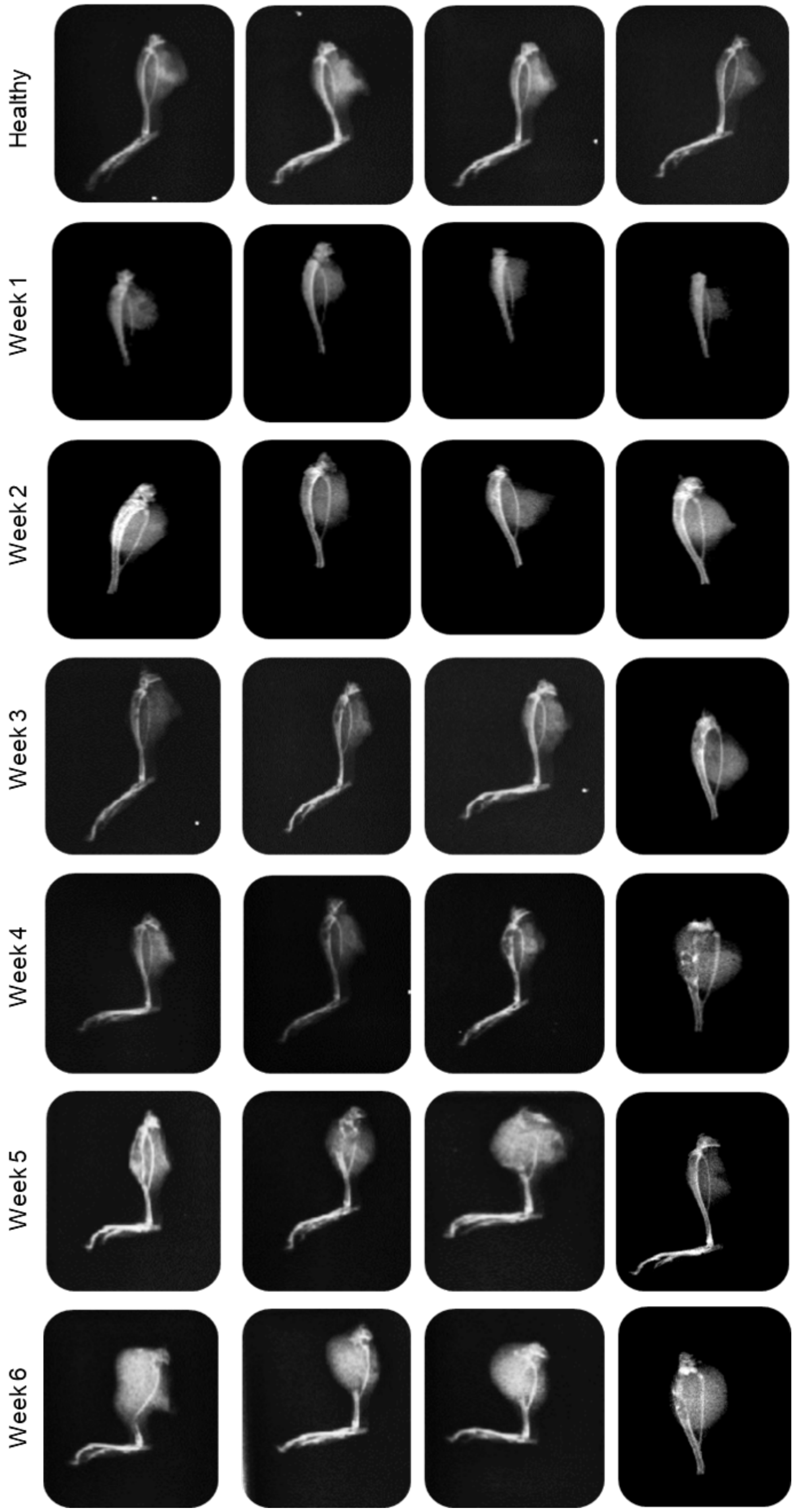

Figure S2. X-ray image data for the 6 weeks of tumor development. Representative images of IVIS Spectrum X-ray data covering 6 weeks of tumor development following the injection of PC3-Luc cells into the left tibia.

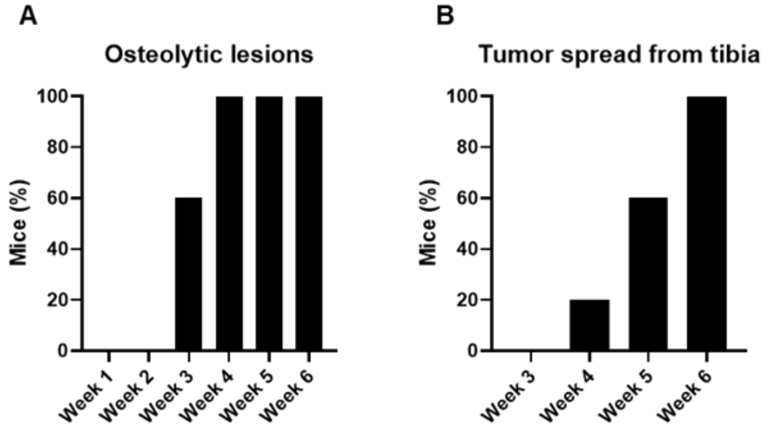

Figure S3. Quantification of osteolytic lesion from X-ray data. A) Percentage of mice with osteolytic lesions in X-ray images from weeks 1 to 6 and **B**) percentage of mice presenting osteolytic lesions that break the tibia (n = 4).

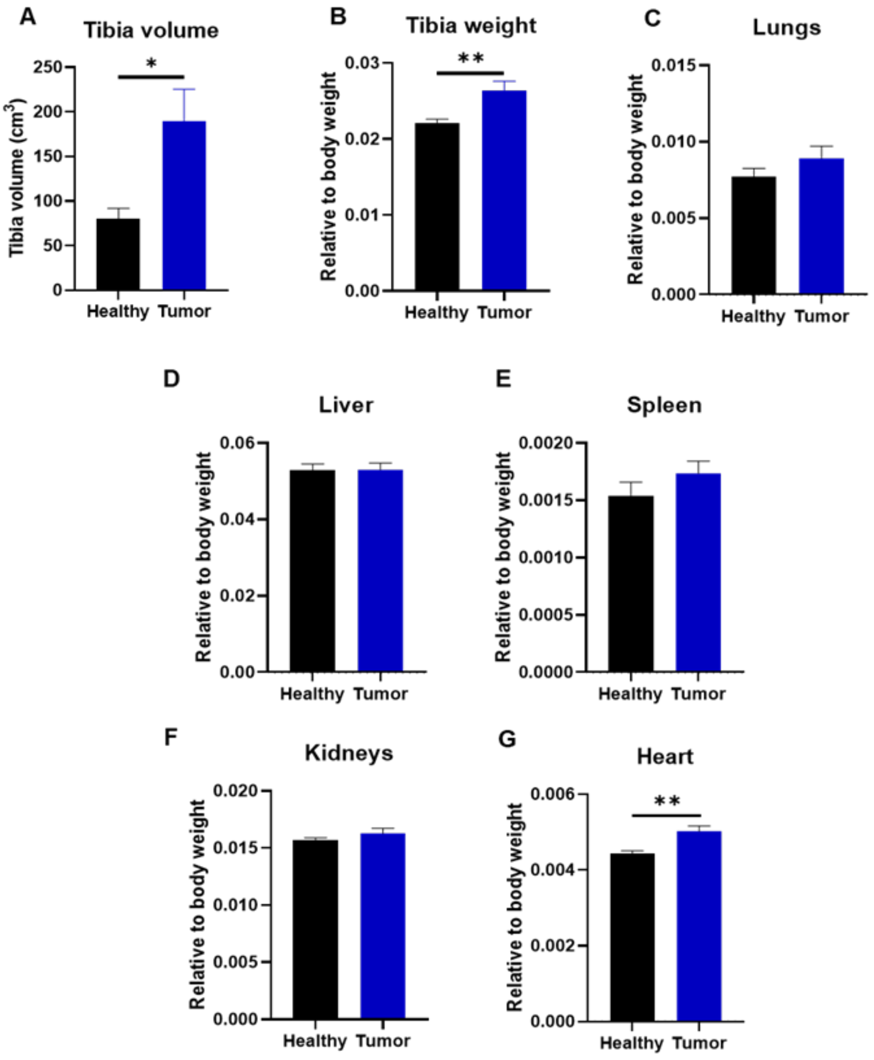

Figure S4. Organ measurements at the experimental endpoint. A) Volume of healthy and tumor-affected tibias (in cm^3) at the experimental endpoint (n = 4). **B-G**) The weight of **B**) healthy and tumor-affected tibias and the **C**) lung, **D**) liver, **E**) spleen, **F**) kidney, and **G**) heart relative to the total body weight of healthy control mice and tumor mice at the experimental endpoint (n ≥ 8). Data expressed as mean±SEM. Statistical analysis performed using ANOVA, *p < 0.05, **p < 0.01.

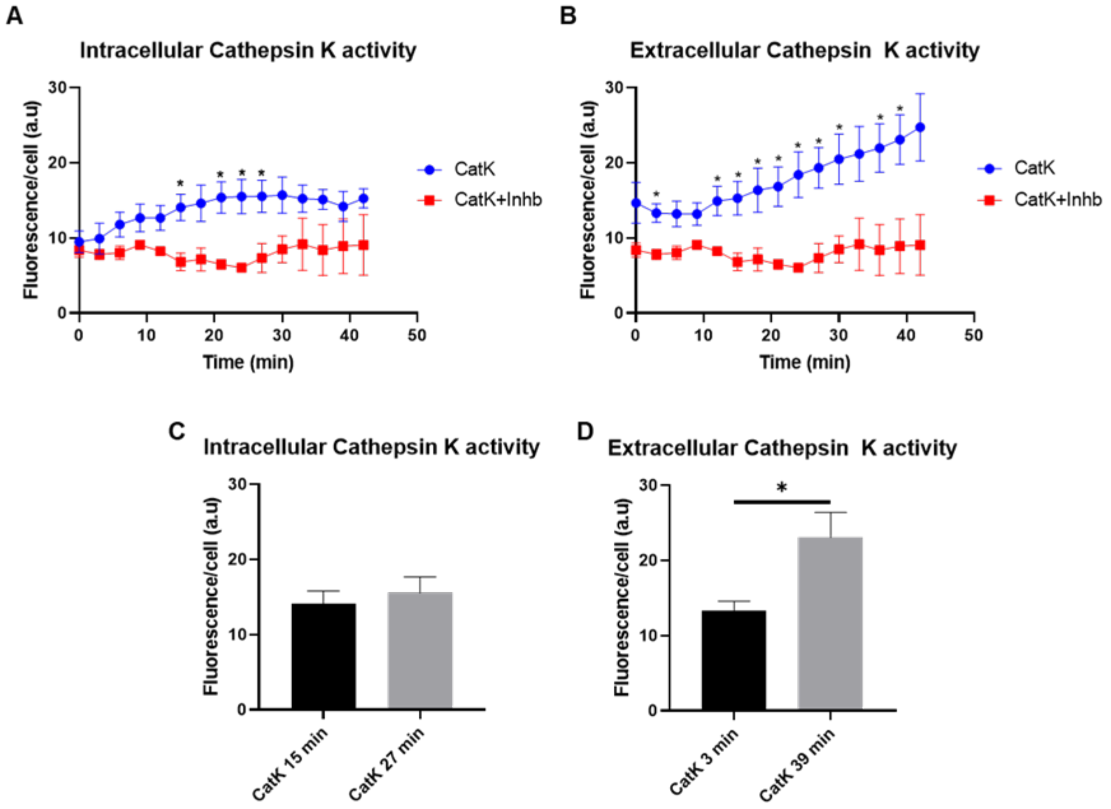

Figure S5. Cathepsin K Activity Determination in PC3-Luc cells. A and **B)** Measurement of **A**) intracellular and **B**) extracellular cathepsin K activity over time measured using a cathepsin K substrate in the presence/absence of a cathepsin K inhibitor (Leupeptin). **C**) Comparison of intracellular cathepsin K activity at the first significant point (15 min) and last significant point (27 min) (n = 3). **D**) Comparison of extracellular cathepsin K activity at the first significant point (3 min) and last significant point (39 min) (n = 3). Abbreviations - A.U.: arbitrary units. Data expressed as mean ± SEM. Statistical analysis performed using ANOVA, *p < 0.05.

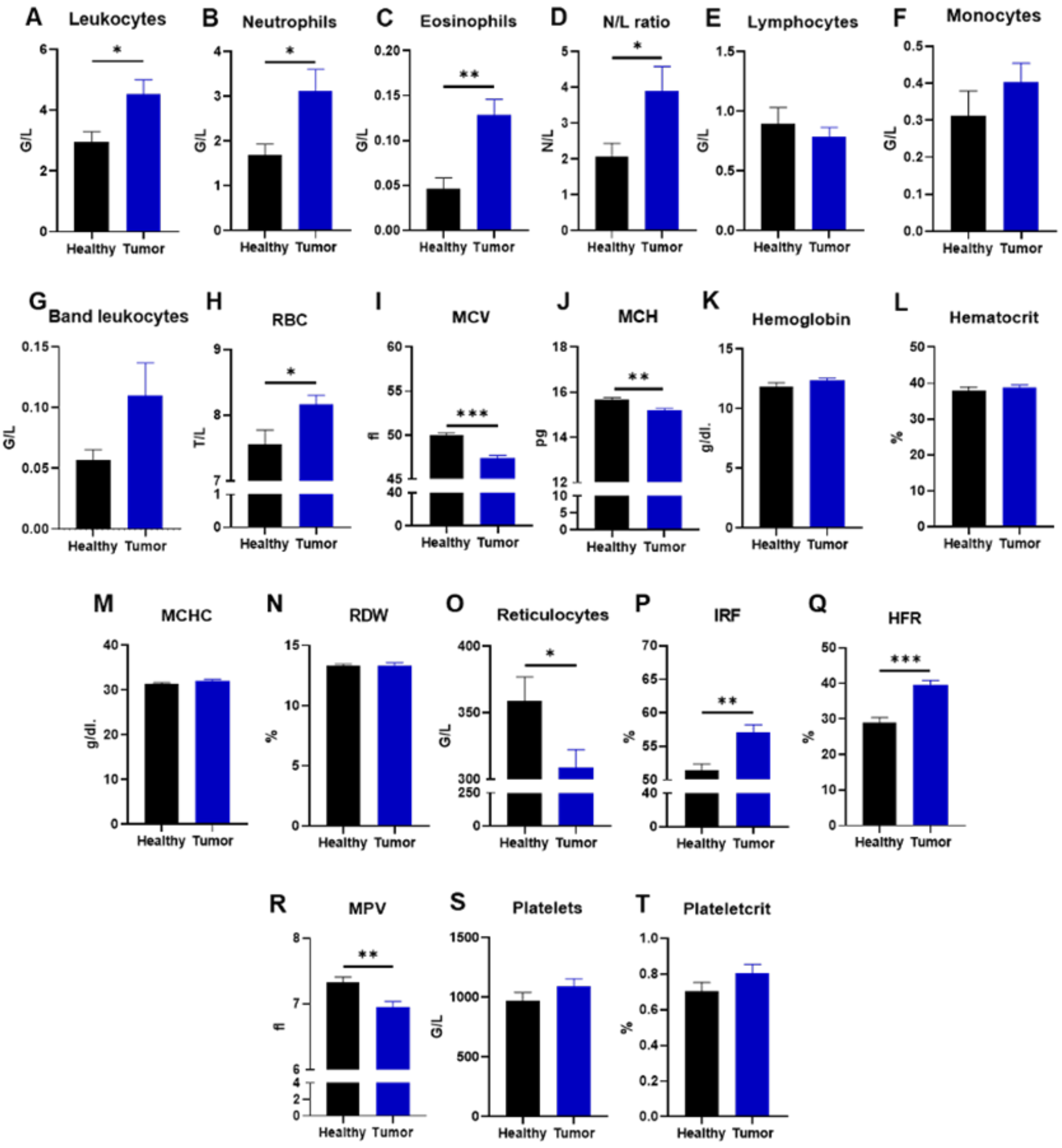

Figure S6. Hematological Analysis of the Bone Metastatic Prostate Cancer Mouse Model at the Experimental Endpoint. Analysis of **A**) leukocytes, **B**) neutrophils, **C**) eosinophils, **D**) the neutrophil-to-lymphocyte ratio (N/L), **E**) lymphocytes, **F**) monocytes, **G**) band leukocytes, **H**) red blood cells (RBC), **I**) mean corpuscular volume (MCV), **J**) mean corpuscular hemoglobin (MCH), **K**) hemoglobin, **L**) hematocrit, **M**) mean corpuscular hemoglobin concentration (MCHC), **N**) red cell distribution width (RDW), **O**) reticulocytes, **P**) immature reticulocyte fraction (IRF), **Q**) the reticulocyte high-fluorescence ratio (HFR), **R**) mean platelet volume (MCV), **S**) platelets and **T**) plateletcrit at the experimental endpoint (week 4). Abbreviations - B: bone; BM: bone marrow; fL: femtoliter; G/L: grams per liter; N/L: neutrophil-to-lymphocyte ratio; pg: pictograms; star: tumor; T/L: tera per liter. Data expressed as mean±SEM (n ≥ 8). Statistical analysis performed using ANOVA, $*p < 0.05$, $**p < 0.01$, $***p < 0.001$.

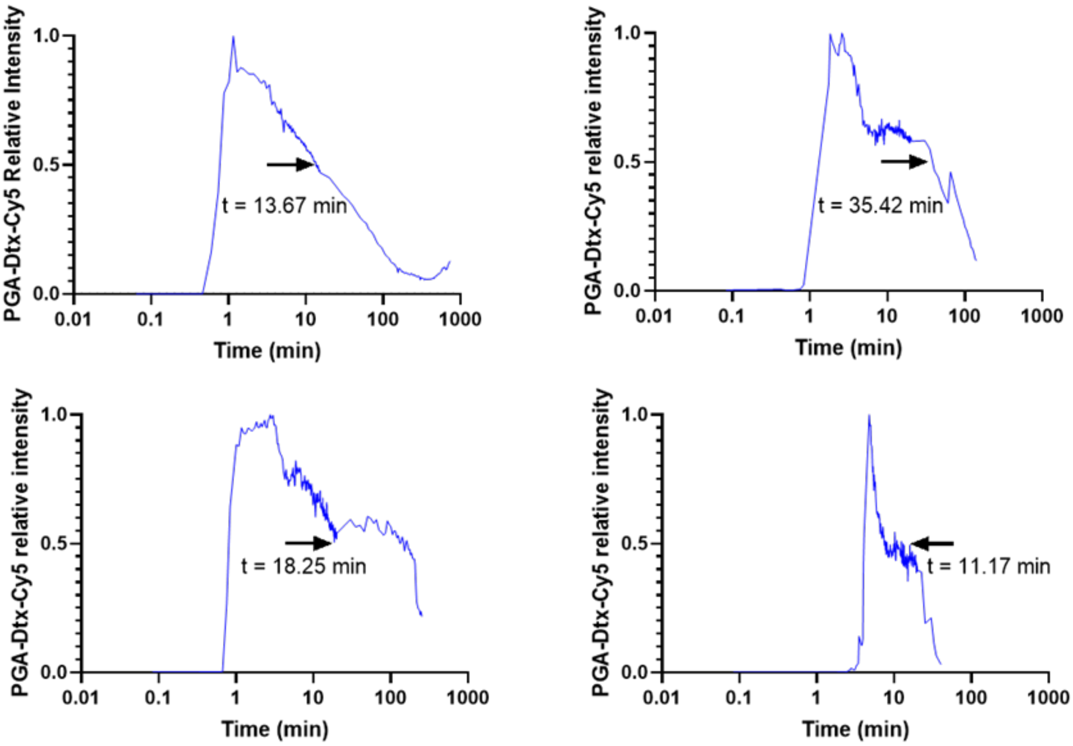

Figure S7. **Circulation Analysis Following Cy5.5-labeled PGA-Dtx Administration.** Fluorescence intensity of the Cy5.5-labeled PGA-Dtx over time in the ear artery in four different mice.

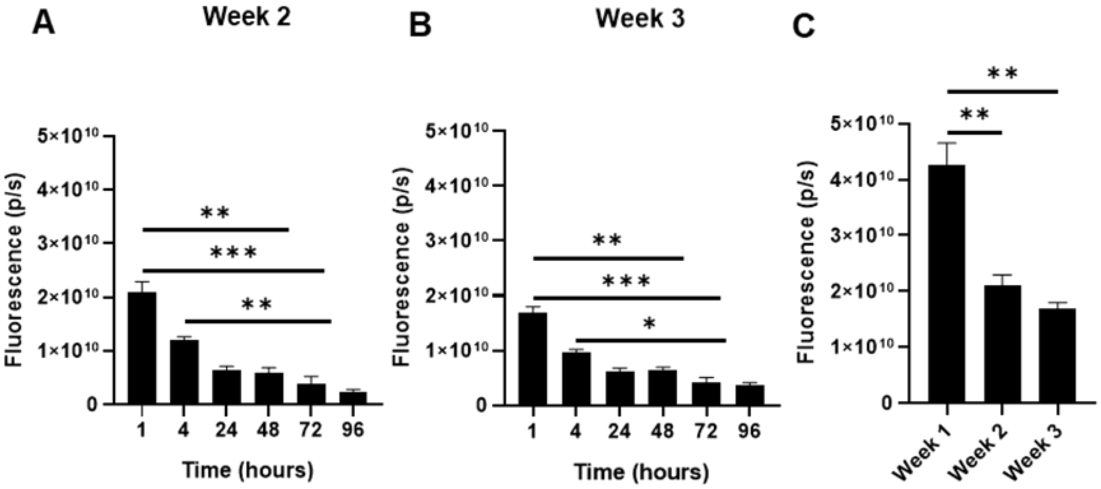

Figure S8. PGA-Dtx Pharmacokinetics. A and B) Relative fluorescence intensity of Cy5.5-labeled PGA-Dtx over time after intravenous injection at **A**) weeks 2 and **B**) 3. **C**) Relative fluorescence intensity of Cy5.5-labeled PGA-Dtx 1 h after intravenous injection at weeks 1, 2, and 3. Data expressed as mean±SEM (n = 6). Statistical analysis performed using ANOVA, *p < 0.05, **p < 0.01, ***p < 0.001.

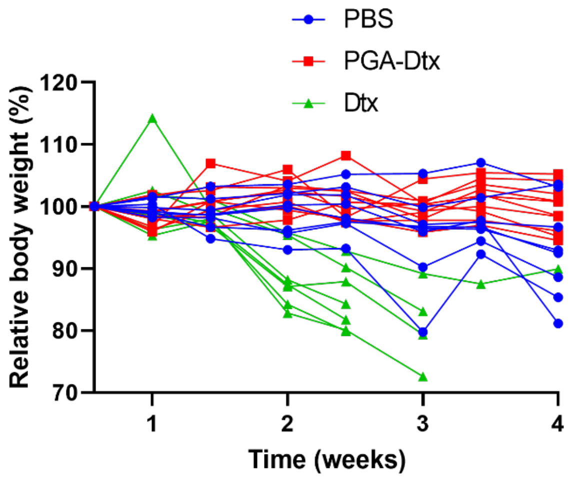

Figure S9. Relative body weight percentage alterations for PBS-, PGA-Dtx and free Dtx-treated individual mice over time (n ≥ 8).

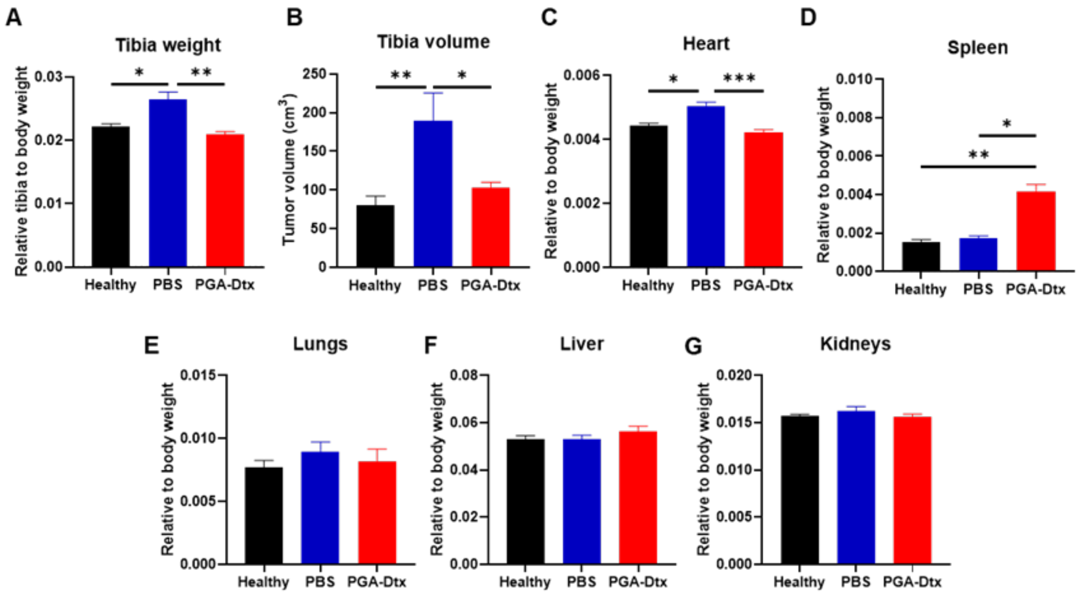

Figure S10. Organ measurements after PGA-Dtx treatment at the experimental endpoint. A) Tumor weight expressed relative to total body weight at week 4 in healthy, PBS-treated, and PGA-Dtx-treated mice. **B**) Tumor volume expressed in cm^3 at week 4 in healthy, PBS-treated, and PGA-Dtx-treated mice. **C**) Heart, **D**) spleen, **E**) lungs, **F**) liver, and **G**) kidney weight expressed relative to total body weight at week 4 in healthy, PBS-treated, and PGA-Dtx-treated mice. Data expressed as mean ± SEM (n ≥ 6). Statistical analysis performed using ANOVA, $^*p < 0.05$, $^{**}p < 0.01$, $^{***}p < 0.001$.

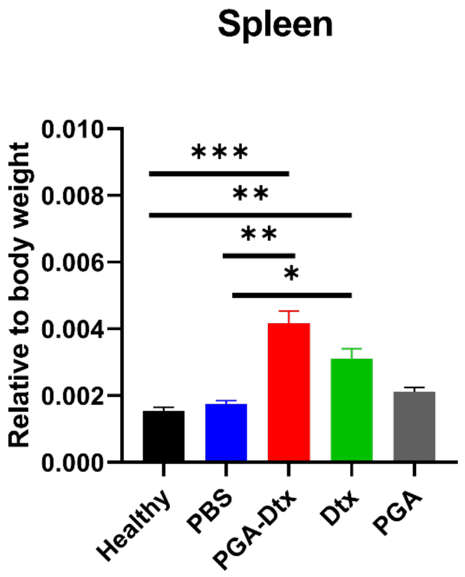

Figure S11. Spleen measurements after treatment. Spleen weight expressed relative to total body weight in healthy, PBS-treated, PGA-Dtx- and PGA-treated mice at experimental endpoint and in free Dtx-treated mice at the time of mouse death. Data expressed as mean ± SEM ($n \geq 6$). Statistical analysis performed using ANOVA, *$p < 0.05$, **$p < 0.01$, ***$p < 0.001$.

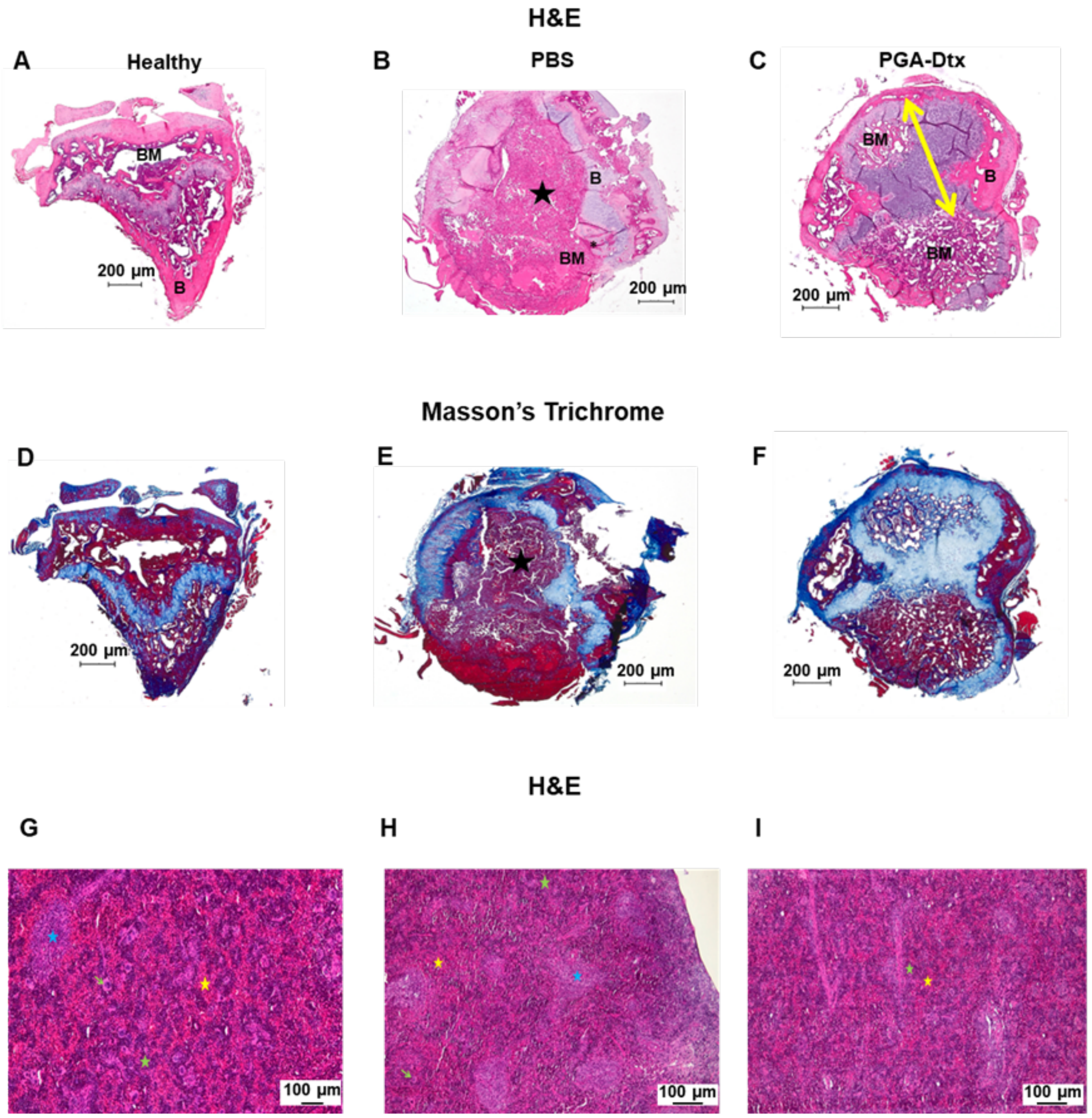

Figure S12. Histological Analysis in the Bone Metastatic Prostate Cancer Mouse Model Following PGA-Dtx Treatment. A-C) Hematoxylin and eosin (H&E) staining of **A**) healthy and tumor-affected tibias of **B**) PBS-treated and **C**) PGA-Dtx-treated mice at the experimental endpoint. **D-F**) Masson's trichrome staining of **D**) healthy and tumor-affected tibias of **E**) PBS-treated and **F**) PGA-Dtx-treated mice at the experimental endpoint (black star: tumor; yellow arrow: chondral metaplasia). **G-I**) H&E staining of the spleen of **G**) healthy mice and **H**) PBS-treated and **I**) PGA-Dtx-treated tumor-bearing mice at the experimental endpoint ($n \geq 3$) (yellow star: red pulp; green star: white pulp; blue star: periarteriolar lymphoid nodule; green arrow: multinucleated giant cells). Abbreviations - B: bone; BM: bone matrix; H&E: hematoxylin and eosin.

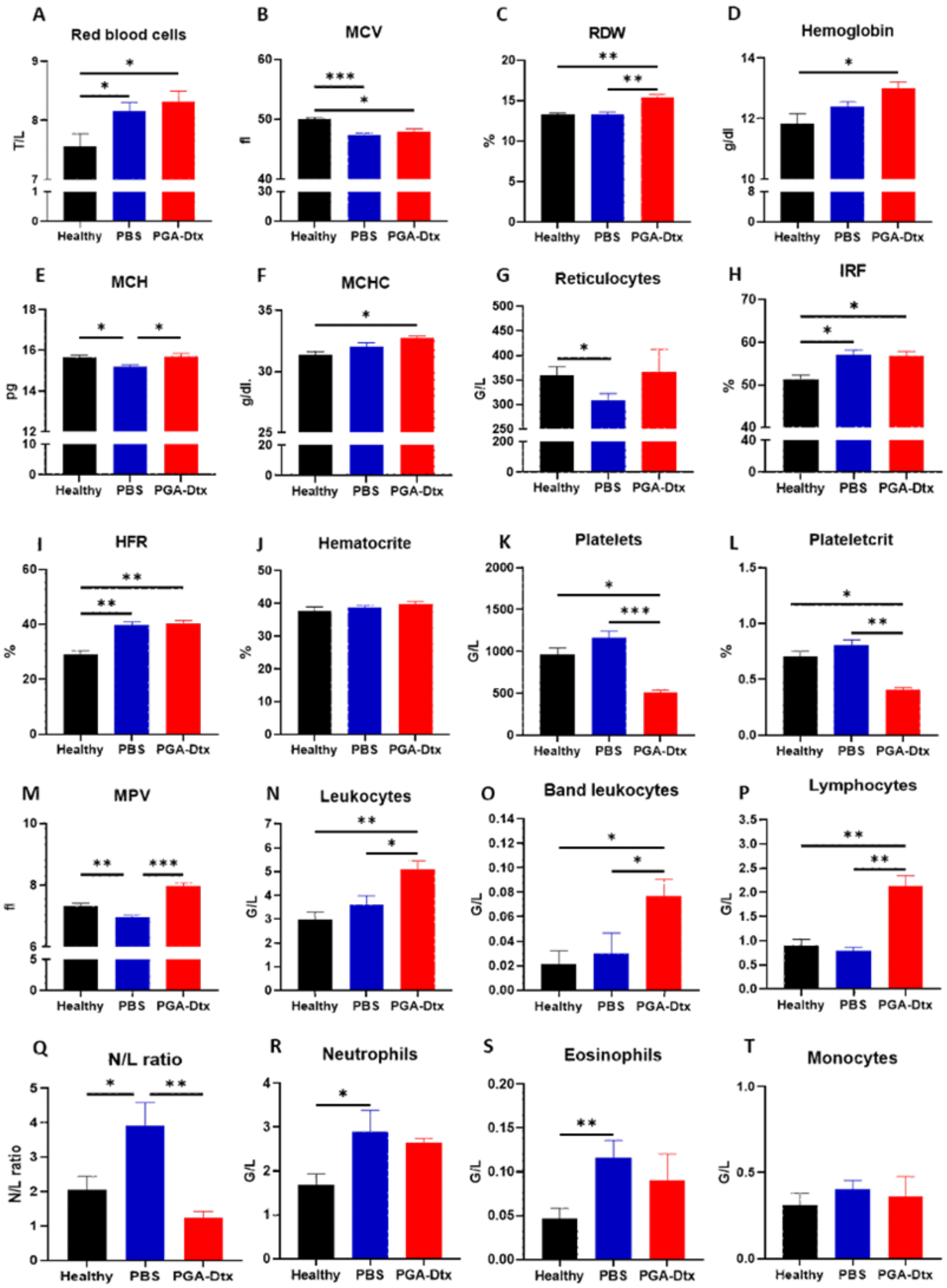

Figure S13. Blood Analysis in the Bone Metastatic Prostate Cancer Mouse Model Following PGA-Dtx Treatment. Levels of **A**) red blood cells, **B**) mean corpuscular volume (MCV), **C**) red cell distribution width (RDW), **D**) hemoglobin, **E**) mean corpuscular hemoglobin, **F**) mean corpuscular hemoglobin concentration (MCHC), **G**) reticulocytes, **H**) immature reticulocyte fraction (IRF), **I**) reticulocyte high-fluorescence ratio (HFR), **J**) hematocrit, **K**) platelets, **L**) plateletcrit, **M**) mean platelet volume (MPV), **N**) leukocytes, **O**) band leukocytes, **P**) lymphocytes, **Q**) neutrophil-to-lymphocyte ratio (N/L ratio), **R**) neutrophils, **S**) eosinophils and **T**) monocytes in healthy and PBS- and PGA-Dtx-treated mice at the experimental endpoint. Abbreviations - %: percentage; fL: femtoliter; g/dL: grams per deciliter; G/L: grams per liter; pg: picograms; T/L: Tera per liter. Data

expressed as mean±SEM (n ≥ 6). Statistical analysis performed using ANOVA, *p < 0.05, **p < 0.01, ***p < 0.001.

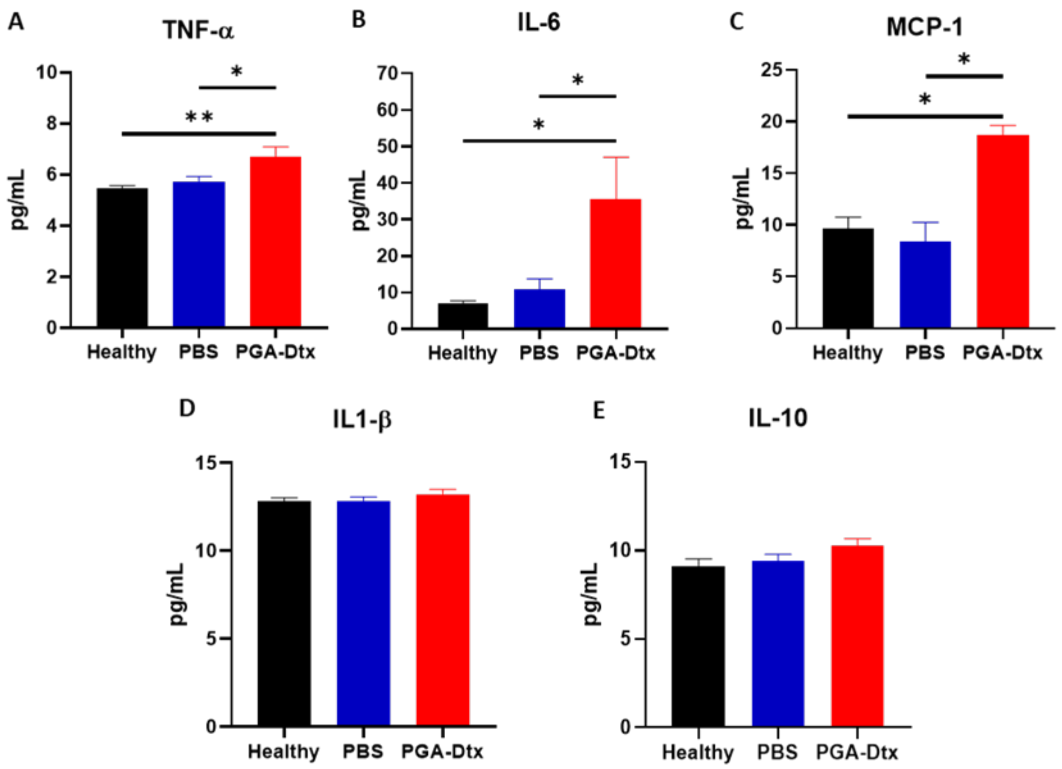

Figure S14. Analysis of Cytokine Levels in the Bone Metastatic Prostate Cancer Mouse Model Following PGA-Dtx Treatment. Serum levels of **A**) TNF-α, **B**) IL-6, **C**) MCP-1, **D**) IL-1β, and **E**) IL-10 in healthy mice and PBS-treated and PGA-Dtx-treated tumor-bearing mice at 4 weeks. Abbreviations - pg/mL: picograms per milliliter. Data expressed as mean ± SEM (n ≥ 5). Statistical analysis performed using ANOVA, *p < 0.05, **p < 0.01.
